# Supplementary figures and images for: Amidase and lysozyme dual functions in TseP reveal a new family of chimeric effectors in the type VI secretion system
Source: eLife. 2025 Mar 10;13:RP101125. doi: 10.7554/eLife.101125 (PMC11893102; doi:10.7554/eLife.101125)

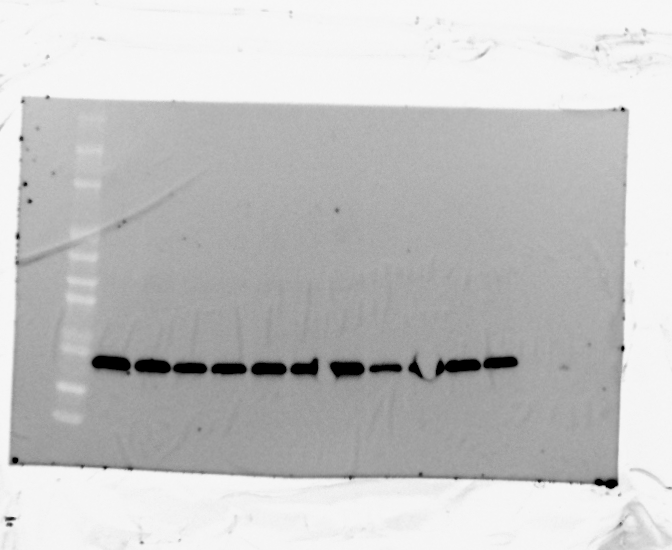

Supplement: Figure 1—source data 1. [file elife-101125-fig1-data1.zip › Figure 1-source data 1/Figure 1A cell lysates Hcp.tif]

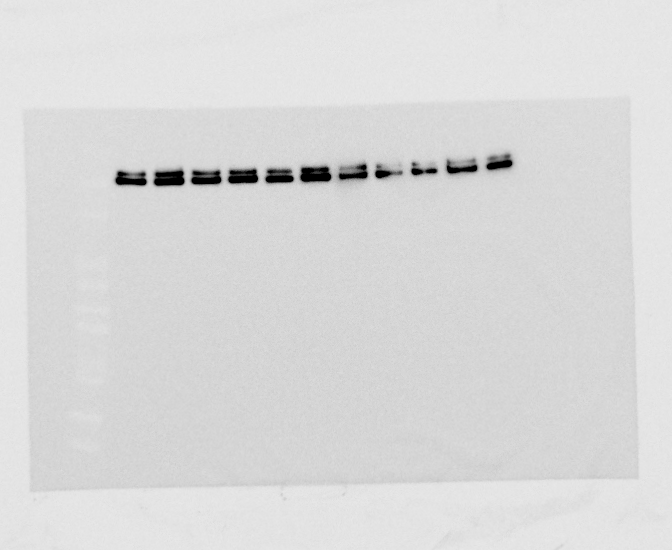

Supplement: Figure 1—source data 1. [file elife-101125-fig1-data1.zip › Figure 1-source data 1/Figure 1A cell lysates RpoB.tif]

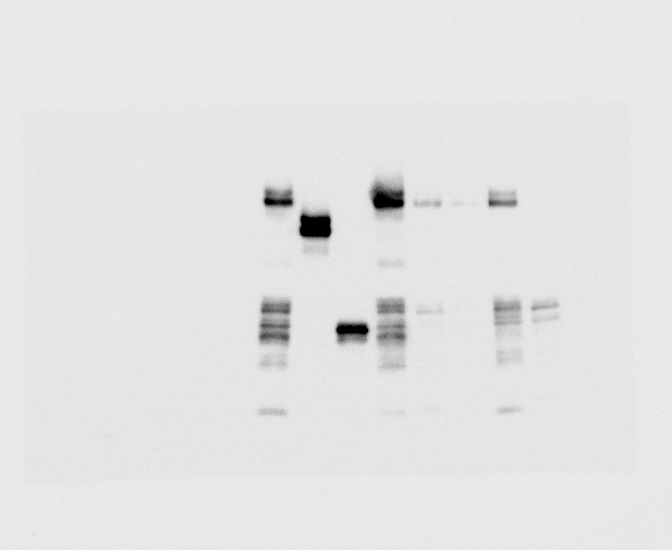

Supplement: Figure 1—source data 1. [file elife-101125-fig1-data1.zip › Figure 1-source data 1/Figure 1A cell lysates v5.tif]

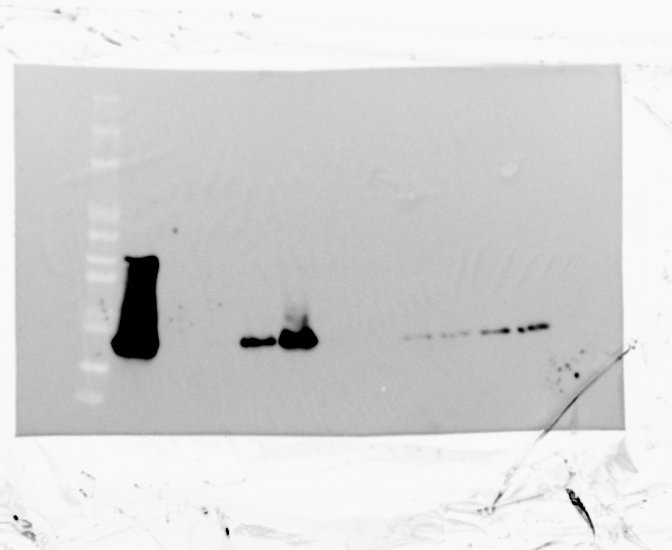

Supplement: Figure 1—source data 1. [file elife-101125-fig1-data1.zip › Figure 1-source data 1/Figure 1A secreted proteins Hcp.tif]

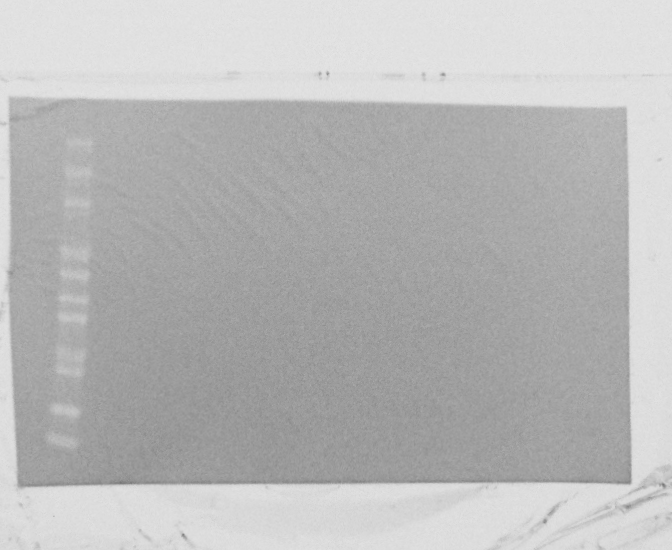

Supplement: Figure 1—source data 1. [file elife-101125-fig1-data1.zip › Figure 1-source data 1/Figure 1A secreted proteins RpoB.tif]

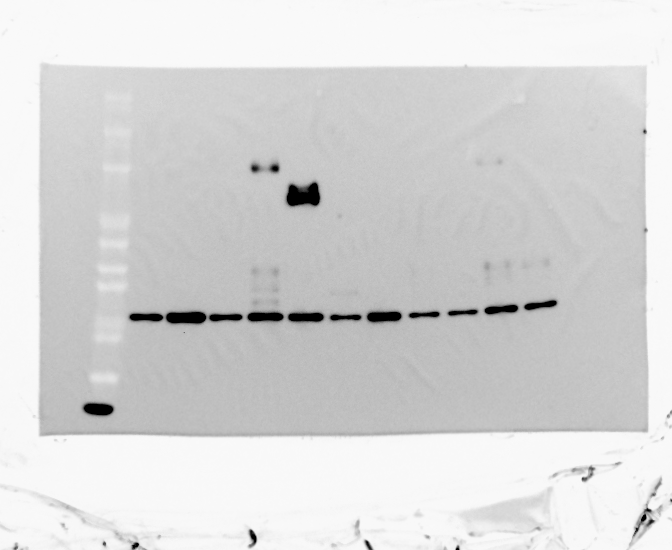

Supplement: Figure 1—source data 1. [file elife-101125-fig1-data1.zip › Figure 1-source data 1/Figure 1A secreted proteins v5.tif]

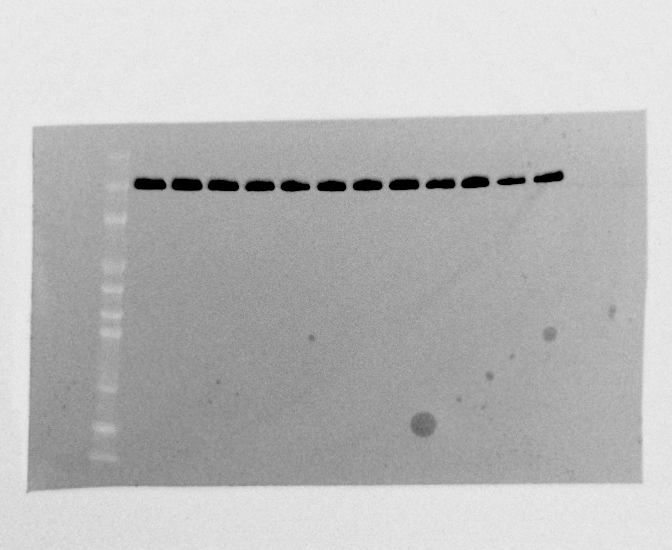

Supplement: Figure 1—source data 1. [file elife-101125-fig1-data1.zip › Figure 1-source data 1/Figure 1D cell lysates RpoB.tif]

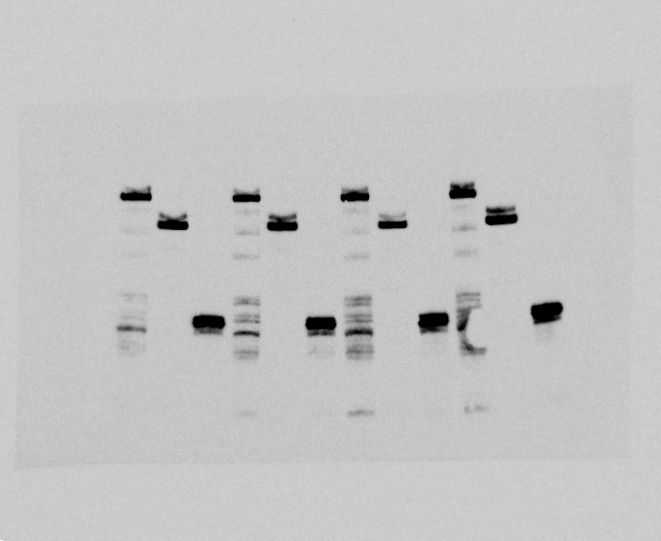

Supplement: Figure 1—source data 1. [file elife-101125-fig1-data1.zip › Figure 1-source data 1/Figure 1D cell lysates v5.tif]

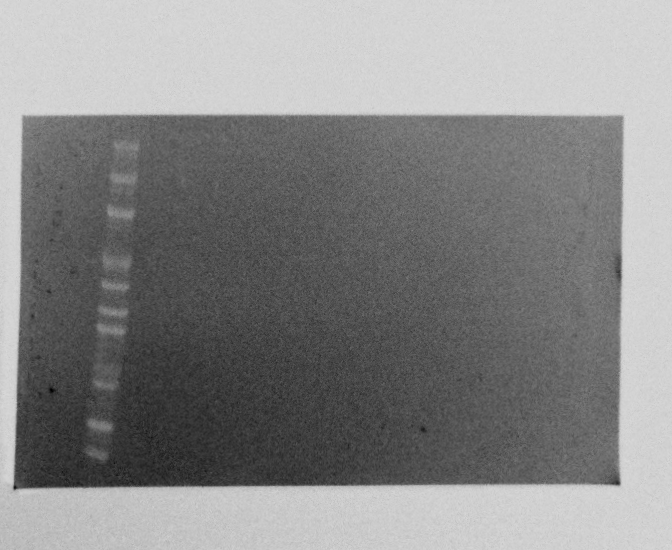

Supplement: Figure 1—source data 1. [file elife-101125-fig1-data1.zip › Figure 1-source data 1/Figure 1D secreted proteins RpoB.tif]

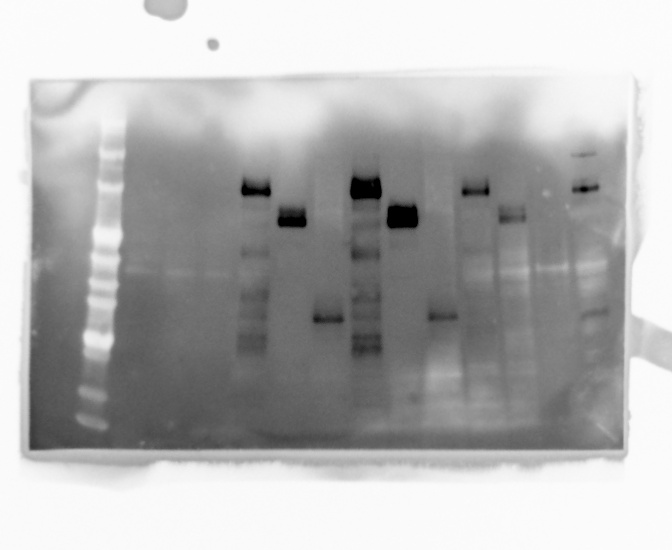

Supplement: Figure 1—source data 1. [file elife-101125-fig1-data1.zip › Figure 1-source data 1/Figure 1D secreted proteins v5.tif]

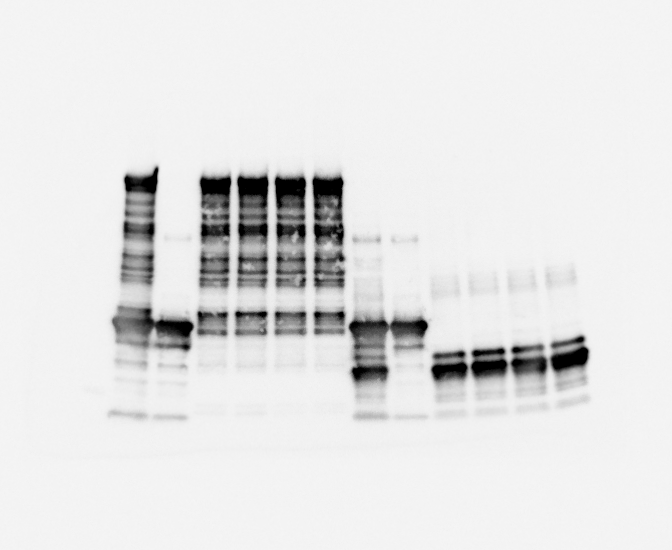

Supplement: Figure 1—source data 1. [file elife-101125-fig1-data1.zip › Figure 1-source data 1/Figure 1E elution His.tif]

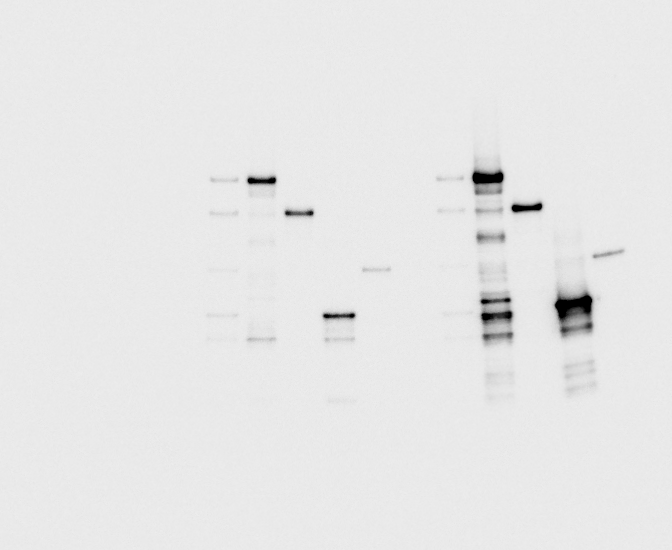

Supplement: Figure 1—source data 1. [file elife-101125-fig1-data1.zip › Figure 1-source data 1/Figure 1E elution v5.tif]

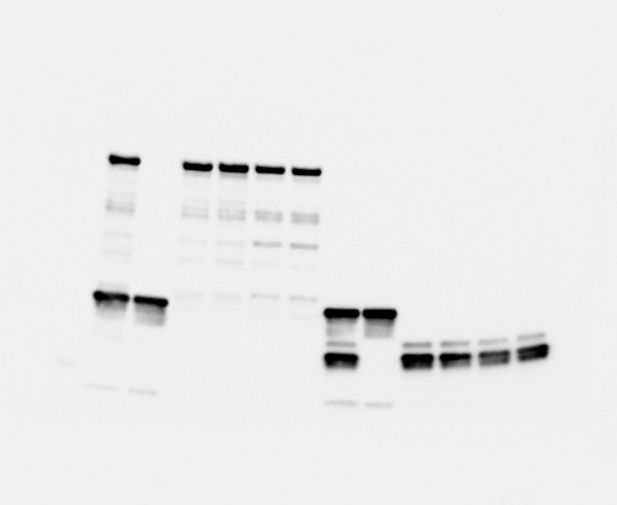

Supplement: Figure 1—source data 1. [file elife-101125-fig1-data1.zip › Figure 1-source data 1/Figure 1E input His.tif]

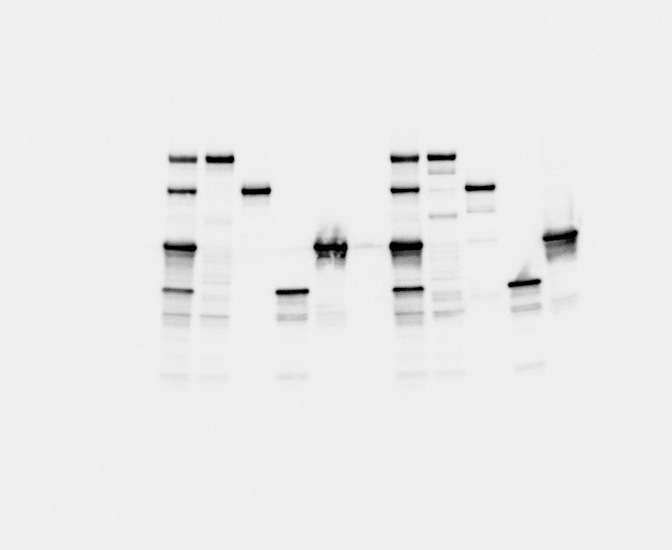

Supplement: Figure 1—source data 1. [file elife-101125-fig1-data1.zip › Figure 1-source data 1/Figure 1E input v5.tif]

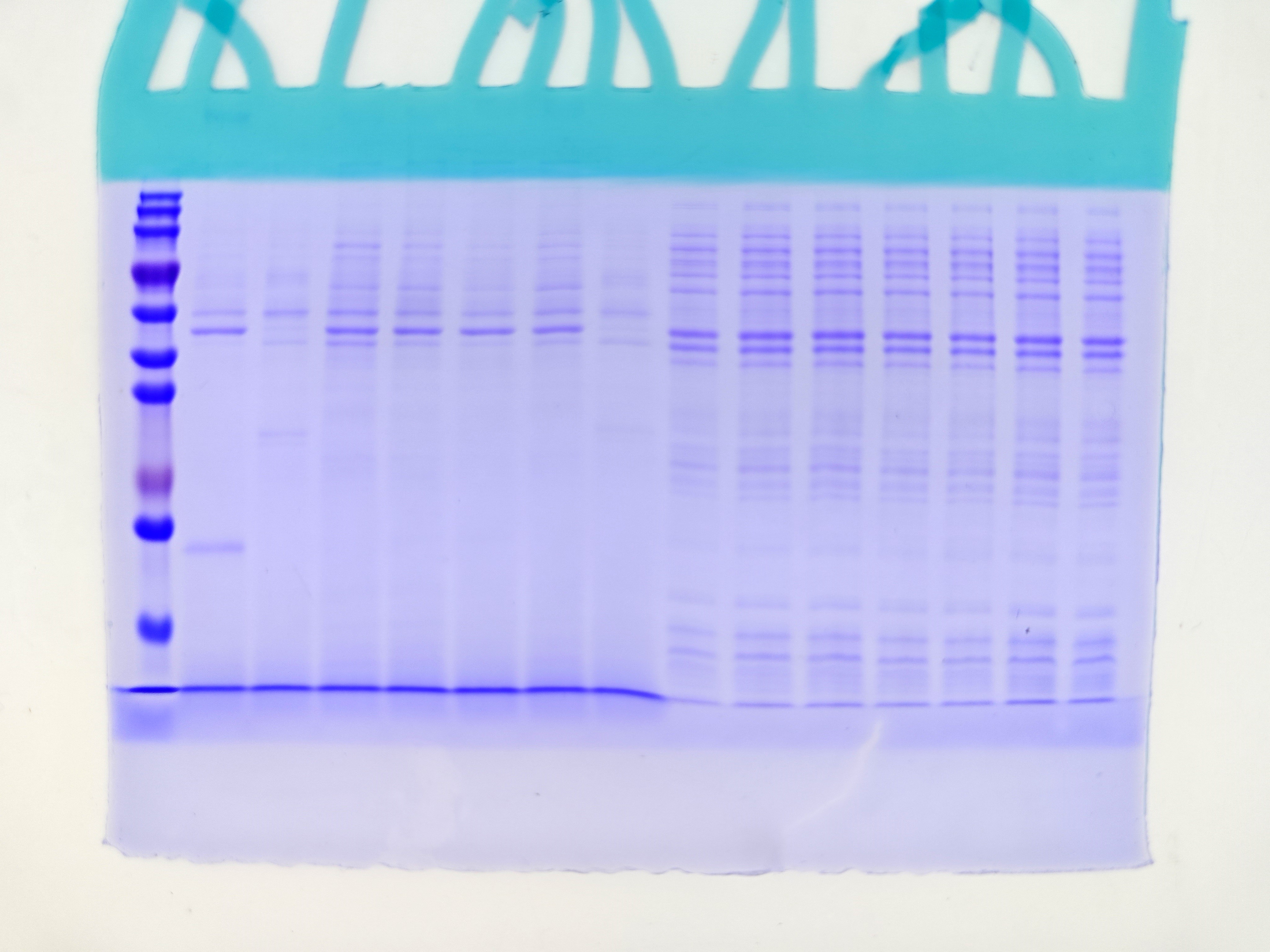

Supplement: Figure 1—figure supplement 1—source data 1. [file elife-101125-fig1-figsupp1-data1.zip › Figure 1-figure supplement 1-source data 1/Figure 1-figure supplement 1.jpg]

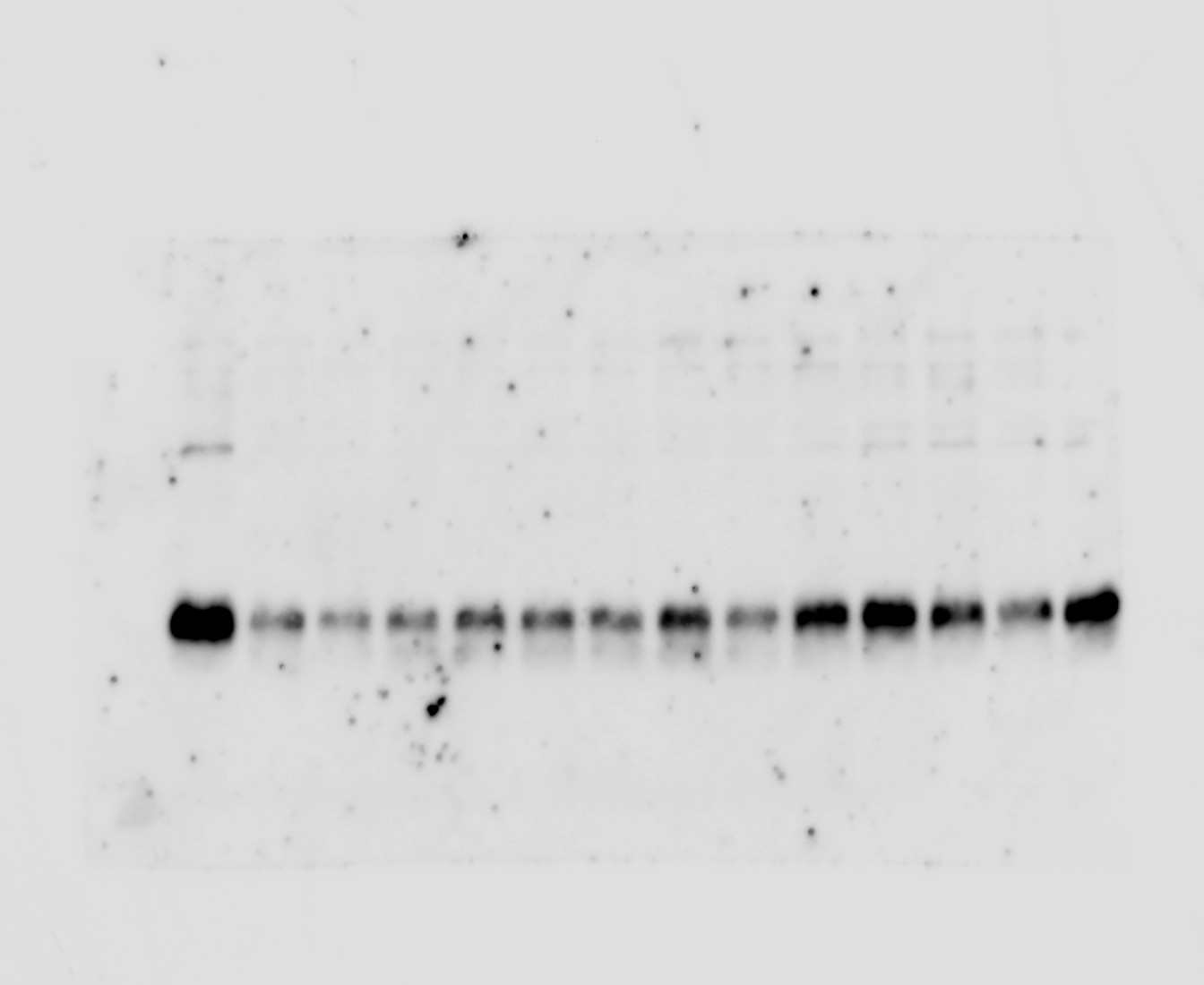

Supplement: Figure 3—source data 1. [file elife-101125-fig3-data1.zip › Figure 3-source data 1/Figure 3A cell lysates Hcp.TIF]

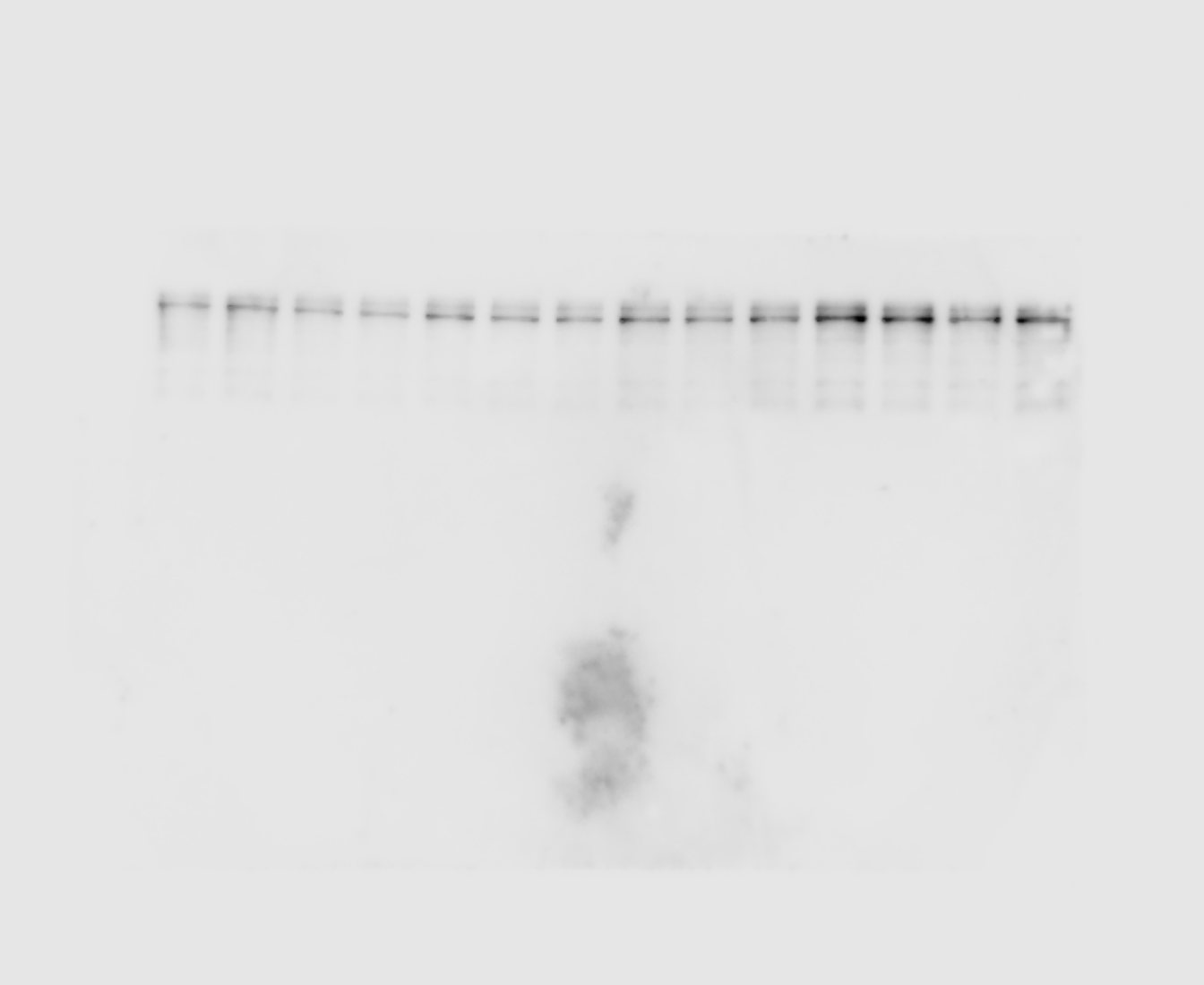

Supplement: Figure 3—source data 1. [file elife-101125-fig3-data1.zip › Figure 3-source data 1/Figure 3A cell lysates RpoB.TIF]

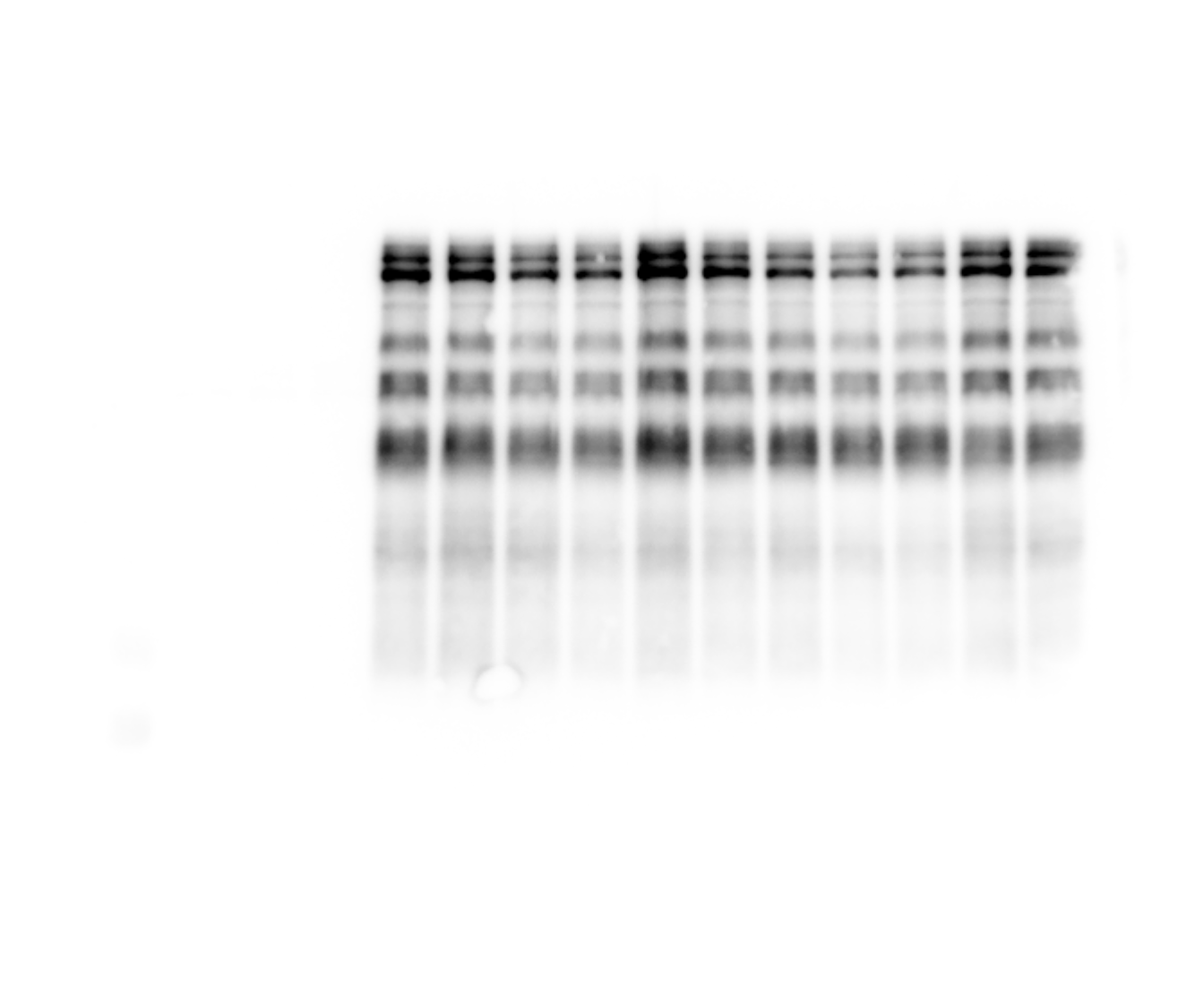

Supplement: Figure 3—source data 1. [file elife-101125-fig3-data1.zip › Figure 3-source data 1/Figure 3A cell lysates v5.TIF]

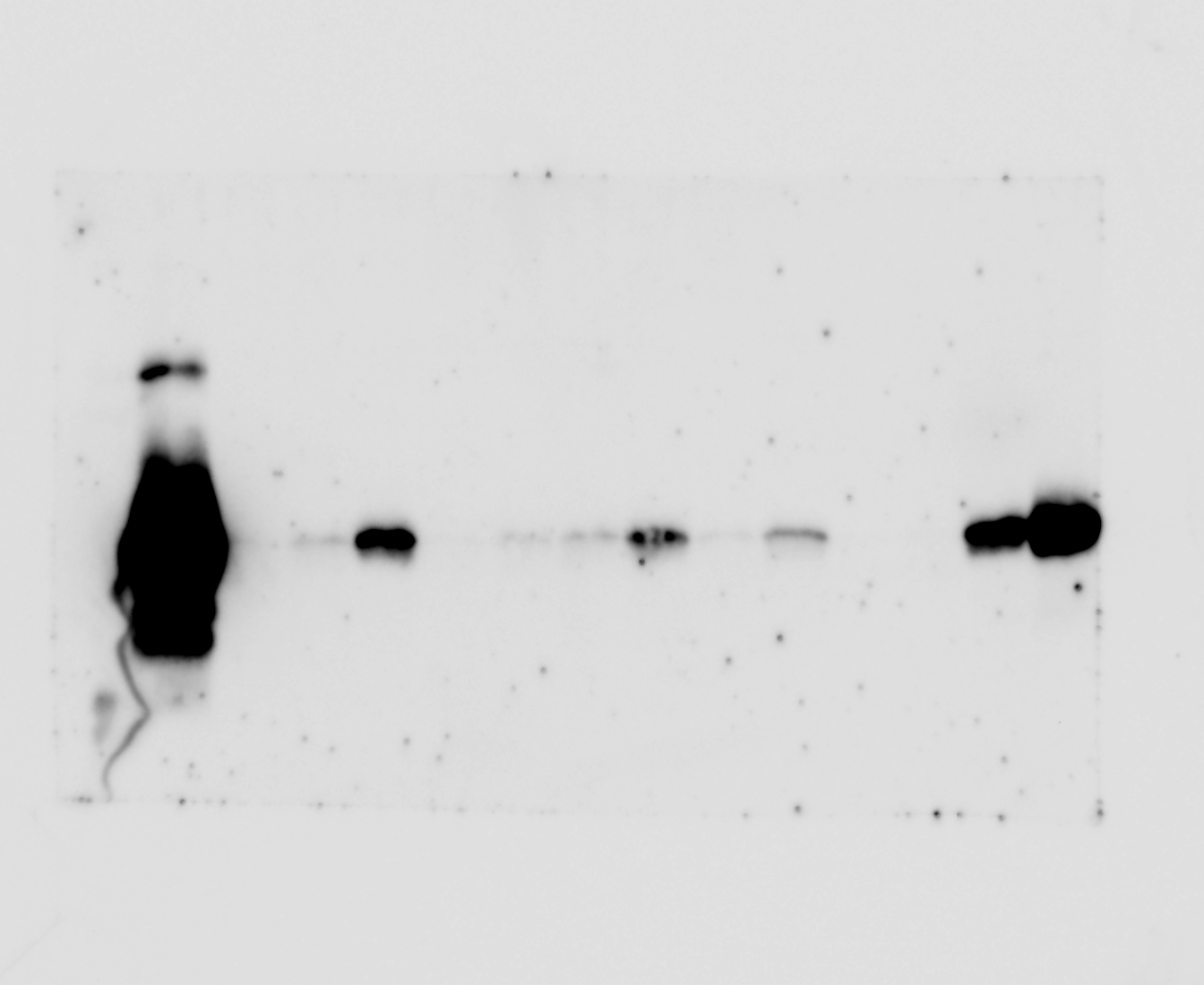

Supplement: Figure 3—source data 1. [file elife-101125-fig3-data1.zip › Figure 3-source data 1/Figure 3A secreted proteins Hcp.TIF]

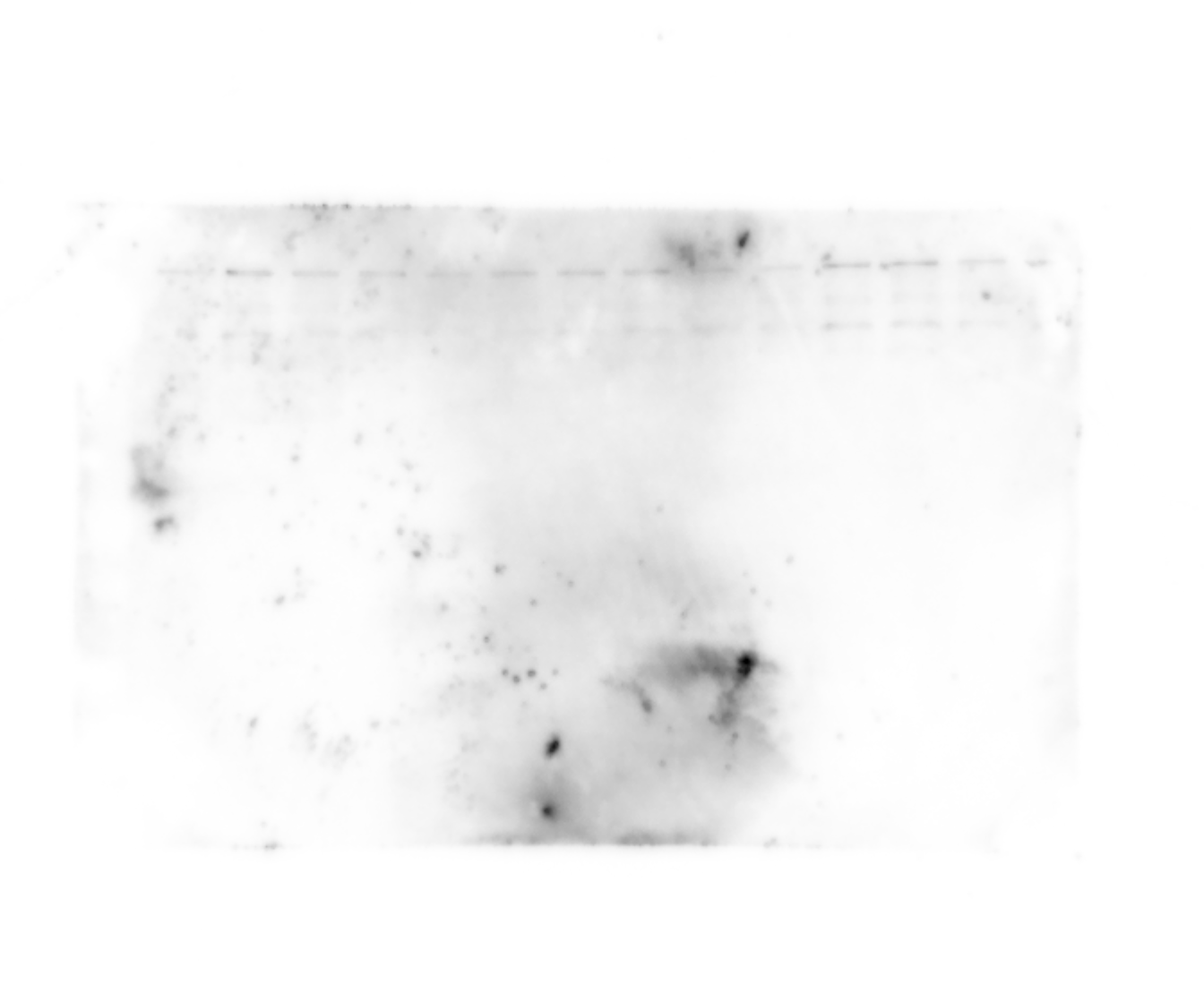

Supplement: Figure 3—source data 1. [file elife-101125-fig3-data1.zip › Figure 3-source data 1/Figure 3A secreted proteins RpoB.TIF]

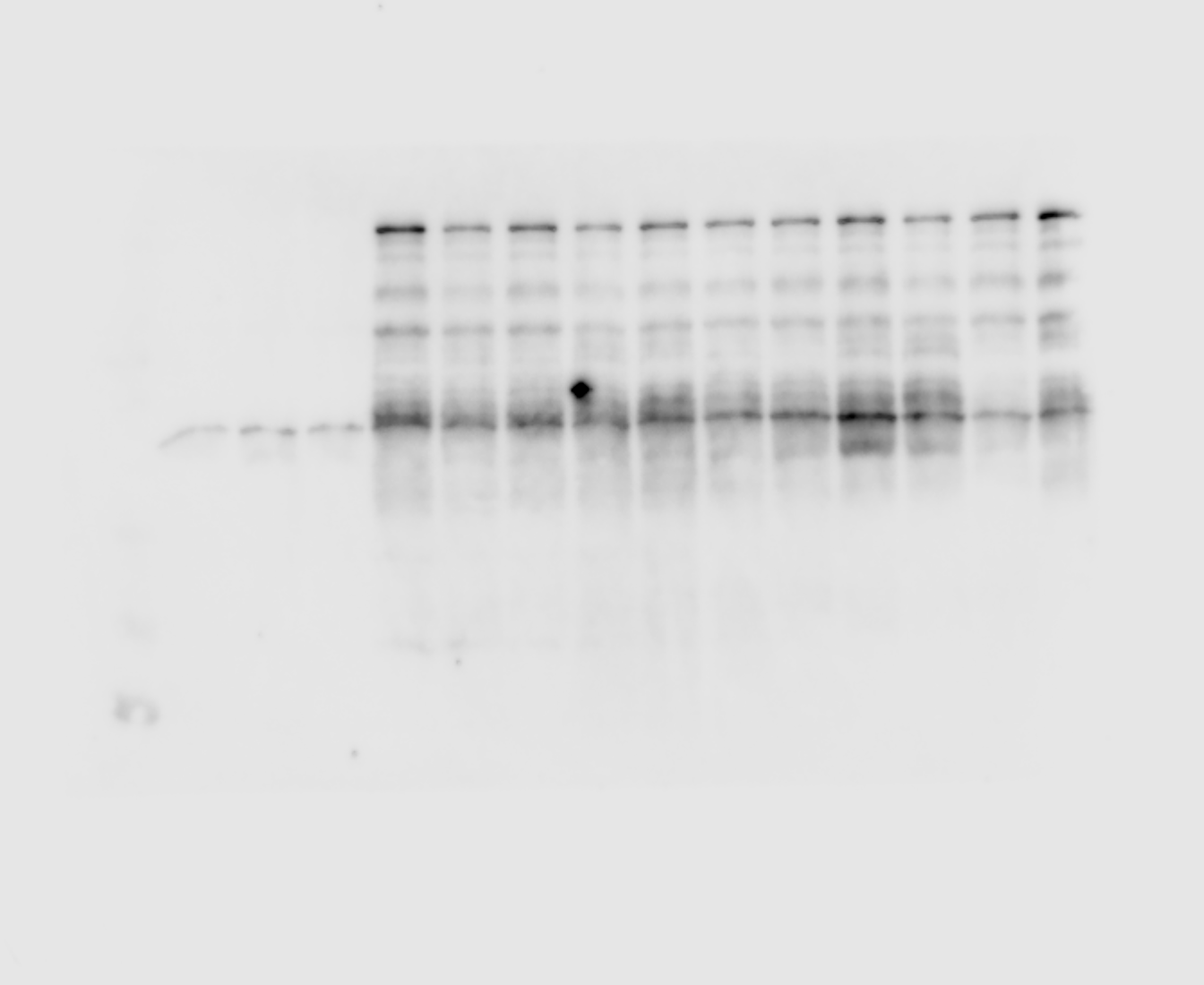

Supplement: Figure 3—source data 1. [file elife-101125-fig3-data1.zip › Figure 3-source data 1/Figure 3A secreted proteins v5.TIF]

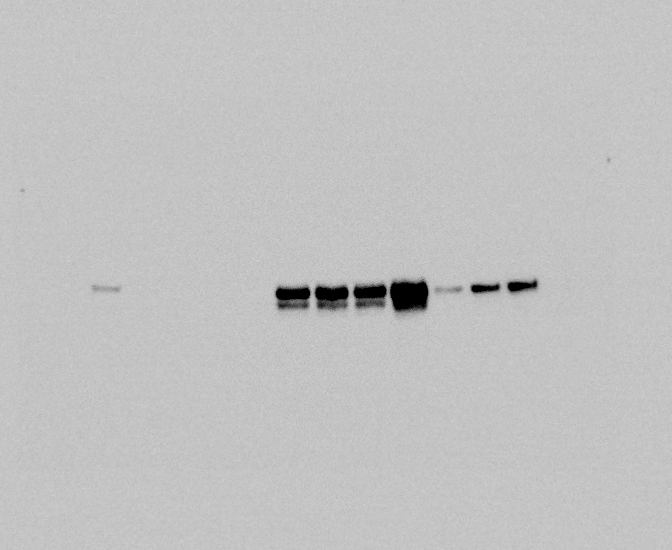

Supplement: Figure 6—source data 1. [file elife-101125-fig6-data1.zip › Figure 6-source data 1/Figure 6E cell lysates v5.tif]

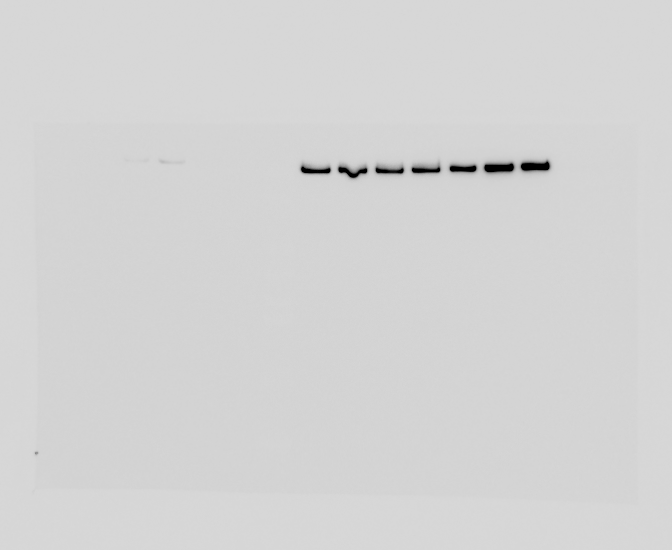

Supplement: Figure 6—source data 1. [file elife-101125-fig6-data1.zip › Figure 6-source data 1/Figure 6E cell lysates-secreted proteins RpoB.tif]

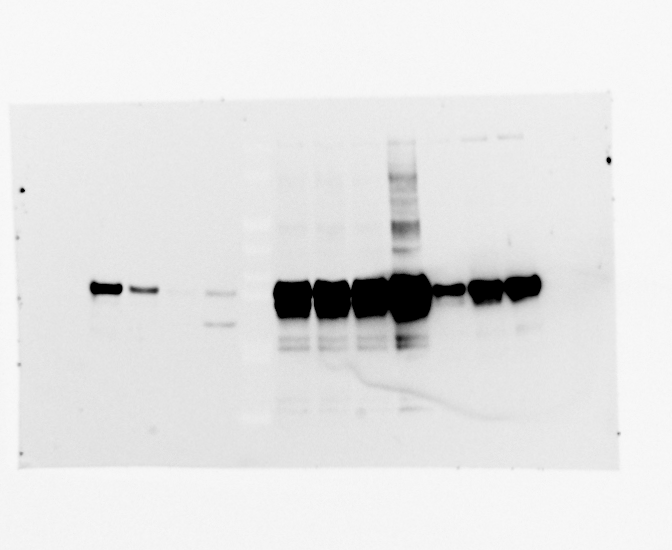

Supplement: Figure 6—source data 1. [file elife-101125-fig6-data1.zip › Figure 6-source data 1/Figure 6E secreted proteins v5.tif]

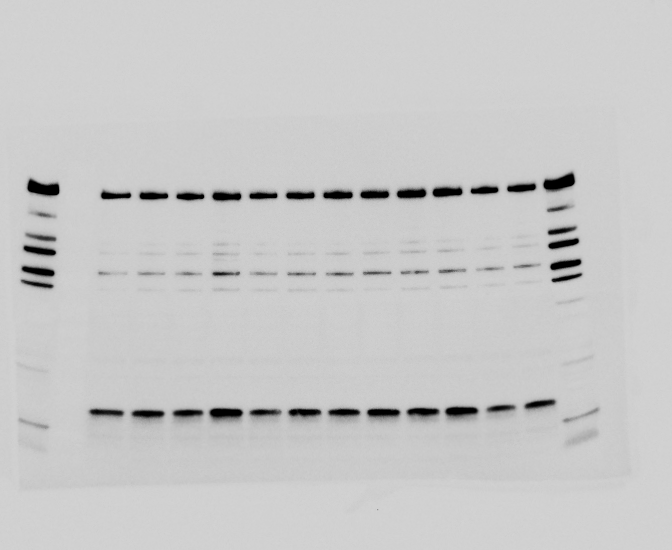

Supplement: Figure 6—figure supplement 2—source data 1. [file elife-101125-fig6-figsupp2-data1.zip › Figure 7-figure supplement 2-source data 1/Figure 7-figure supplement 2A cell lysates RpoB-Hcp.tif]

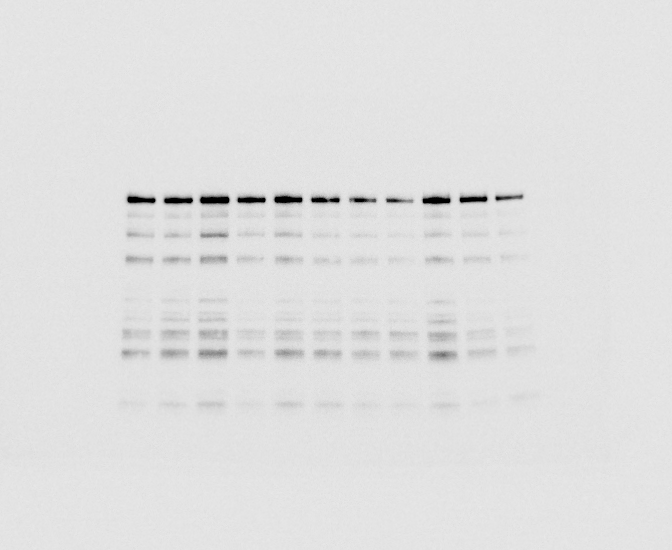

Supplement: Figure 6—figure supplement 2—source data 1. [file elife-101125-fig6-figsupp2-data1.zip › Figure 7-figure supplement 2-source data 1/Figure 7-figure supplement 2A cell lysates v5.tif]

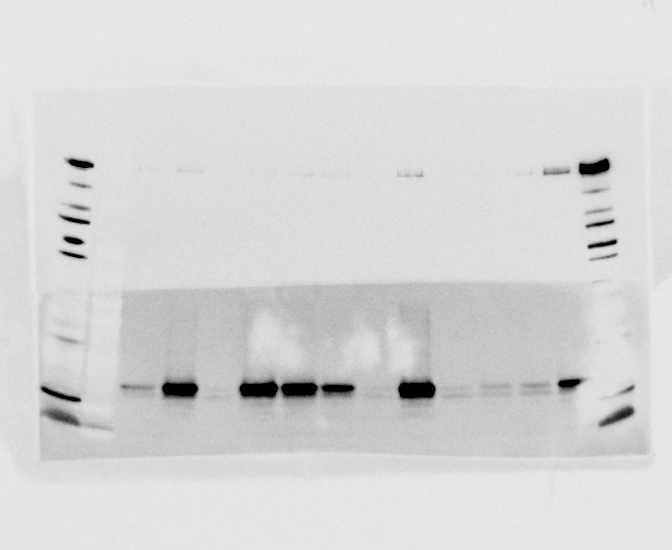

Supplement: Figure 6—figure supplement 2—source data 1. [file elife-101125-fig6-figsupp2-data1.zip › Figure 7-figure supplement 2-source data 1/Figure 7-figure supplement 2A secreted proteins RpoB.tif]

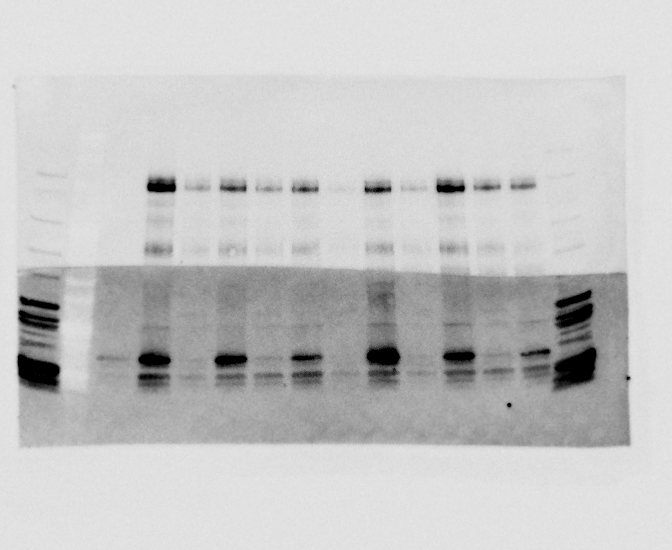

Supplement: Figure 6—figure supplement 2—source data 1. [file elife-101125-fig6-figsupp2-data1.zip › Figure 7-figure supplement 2-source data 1/Figure 7-figure supplement 2A secreted proteins v5-Hcp .tif]

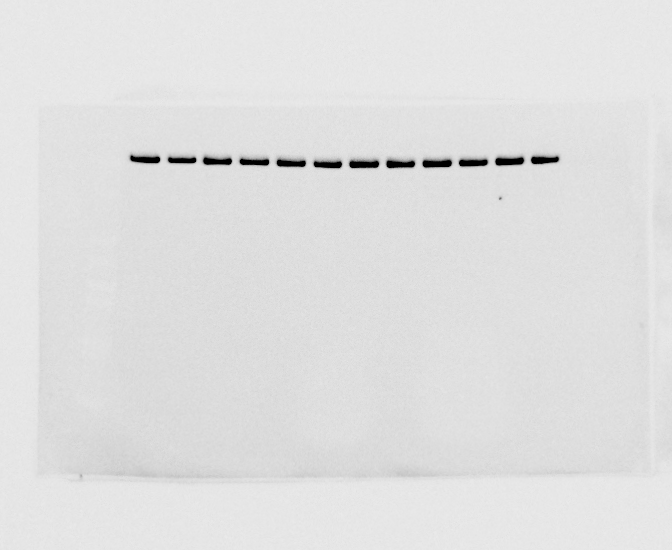

Supplement: Figure 6—figure supplement 2—source data 1. [file elife-101125-fig6-figsupp2-data1.zip › Figure 7-figure supplement 2-source data 1/Figure 7-figure supplement 2B DH5alpha RpoB-1 .tif]

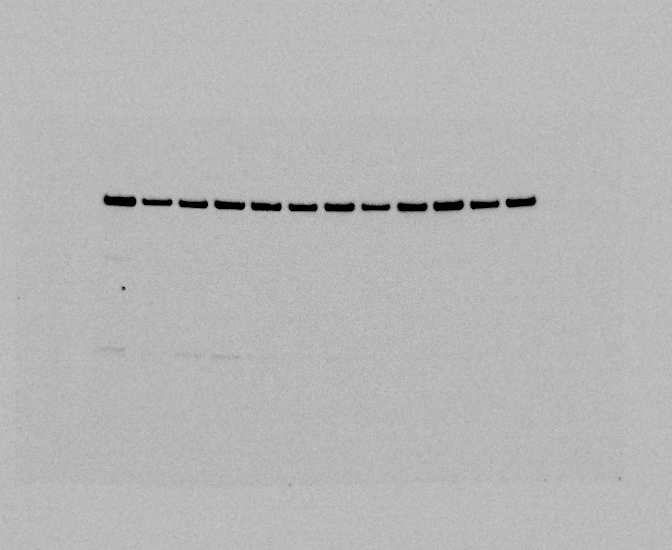

Supplement: Figure 6—figure supplement 2—source data 1. [file elife-101125-fig6-figsupp2-data1.zip › Figure 7-figure supplement 2-source data 1/Figure 7-figure supplement 2B DH5alpha v5-1 .tif]

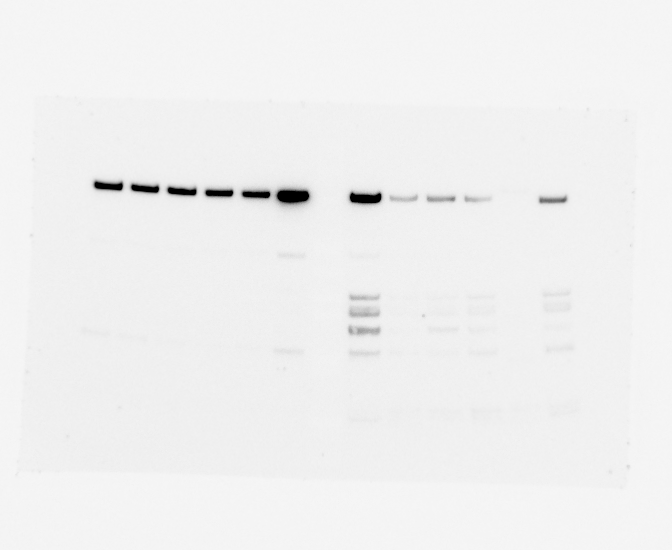

Supplement: Figure 6—figure supplement 2—source data 1. [file elife-101125-fig6-figsupp2-data1.zip › Figure 7-figure supplement 2-source data 1/Figure 7-figure supplement 2B DH5alpha v5-2.tif]

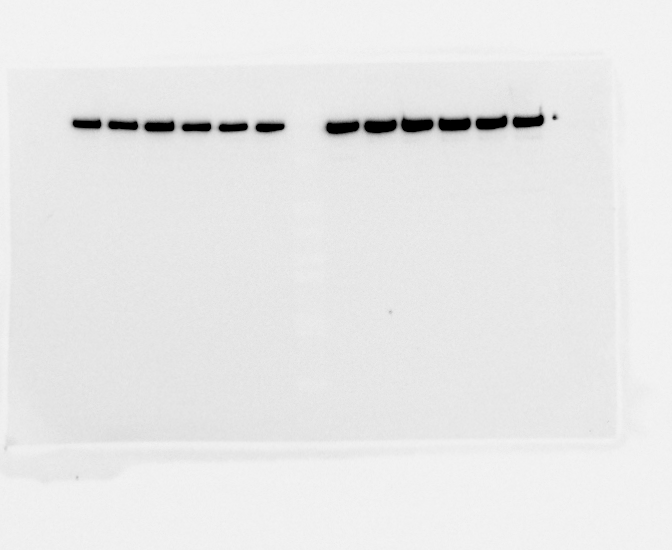

Supplement: Figure 6—figure supplement 2—source data 1. [file elife-101125-fig6-figsupp2-data1.zip › Figure 7-figure supplement 2-source data 1/Figure 7-figure supplement 2B DH5alpha-SSU RpoB-2.tif]

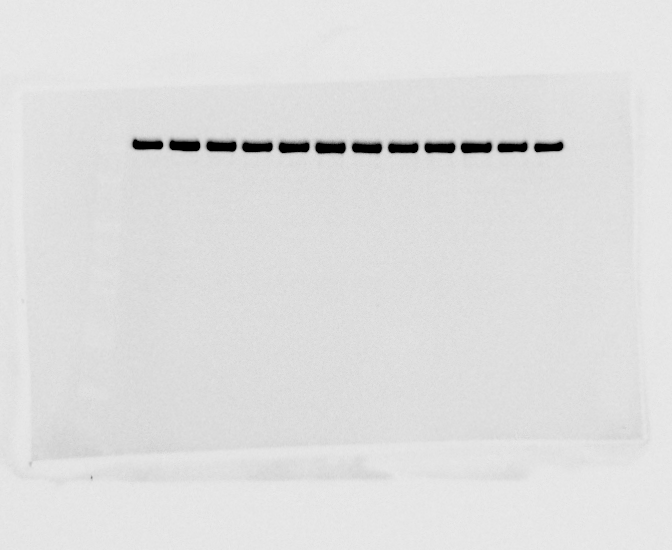

Supplement: Figure 6—figure supplement 2—source data 1. [file elife-101125-fig6-figsupp2-data1.zip › Figure 7-figure supplement 2-source data 1/Figure 7-figure supplement 2B SSU RpoB-1 .tif]

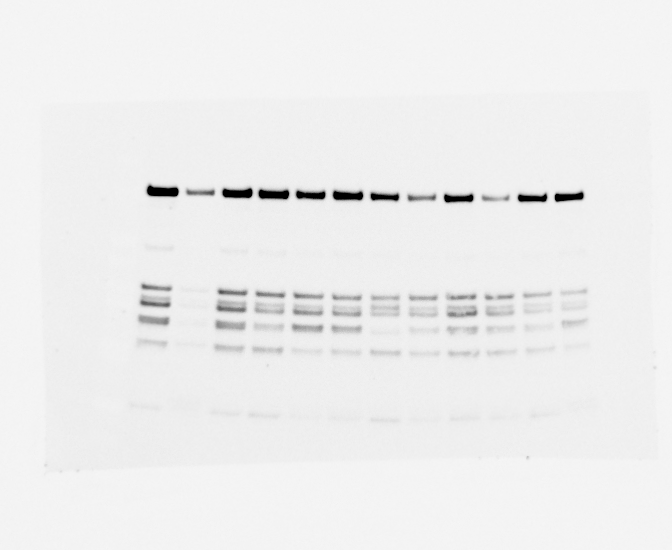

Supplement: Figure 6—figure supplement 2—source data 1. [file elife-101125-fig6-figsupp2-data1.zip › Figure 7-figure supplement 2-source data 1/Figure 7-figure supplement 2B SSU v5-1 .tif]

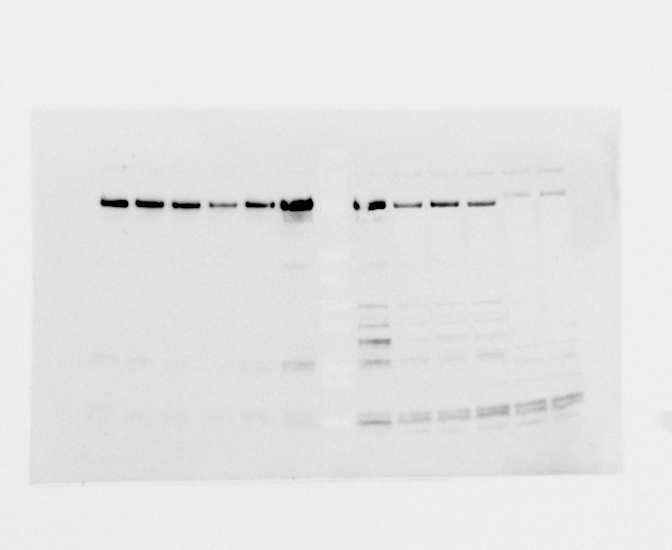

Supplement: Figure 6—figure supplement 2—source data 1. [file elife-101125-fig6-figsupp2-data1.zip › Figure 7-figure supplement 2-source data 1/Figure 7-figure supplement 2B SSU v5-12.tif]

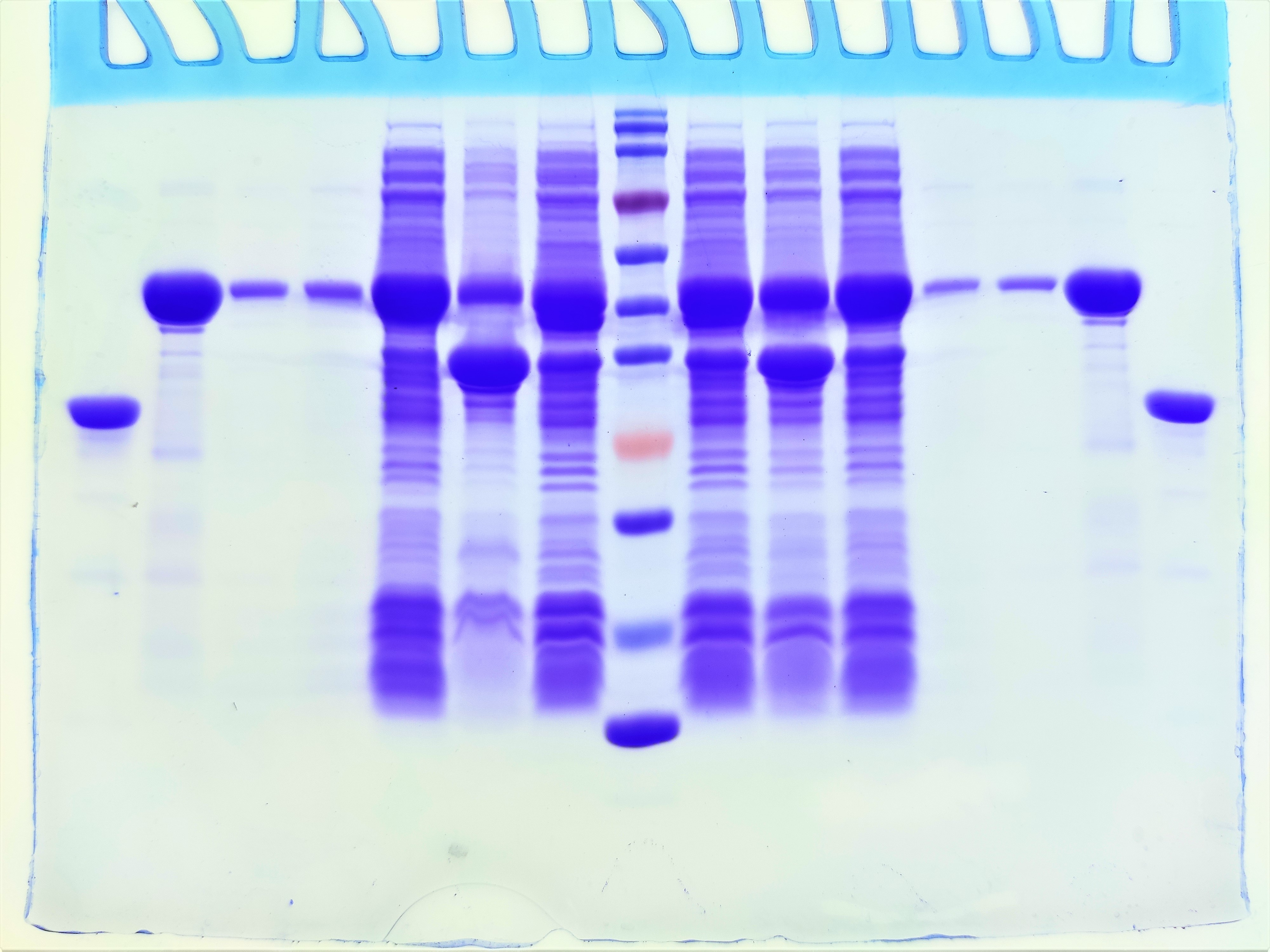

Supplement: Figure 6—figure supplement 3—source data 1. [file elife-101125-fig6-figsupp3-data1.zip › Figure 7-figure supplement 3-source data 1/Figure 7-figure supplement 3-source data 1A-TsePC-TsePC_E663A.jpg]

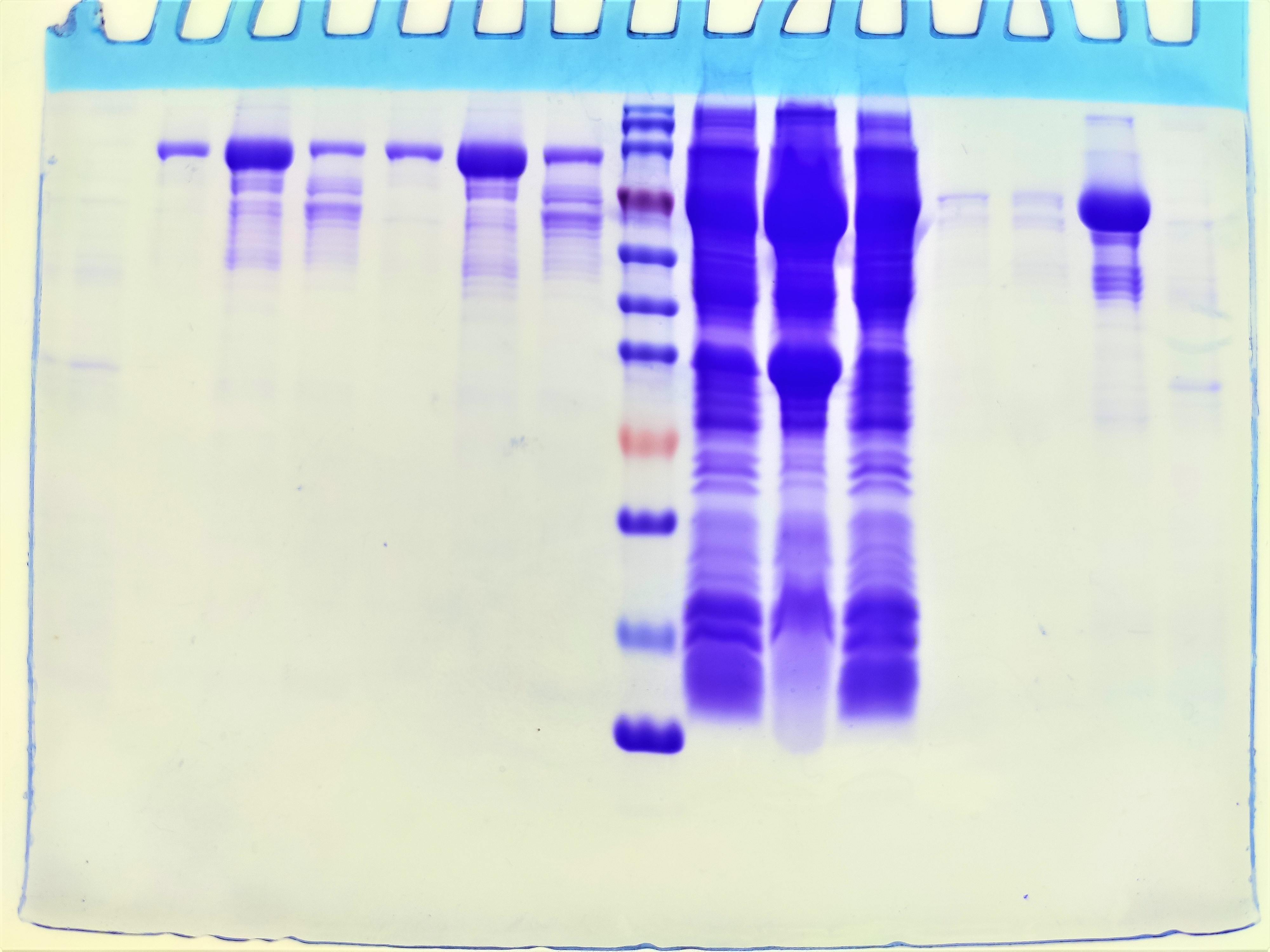

Supplement: Figure 6—figure supplement 3—source data 1. [file elife-101125-fig6-figsupp3-data1.zip › Figure 7-figure supplement 3-source data 1/Figure 7-figure supplement 3-source data 1A-TsePN.jpg]

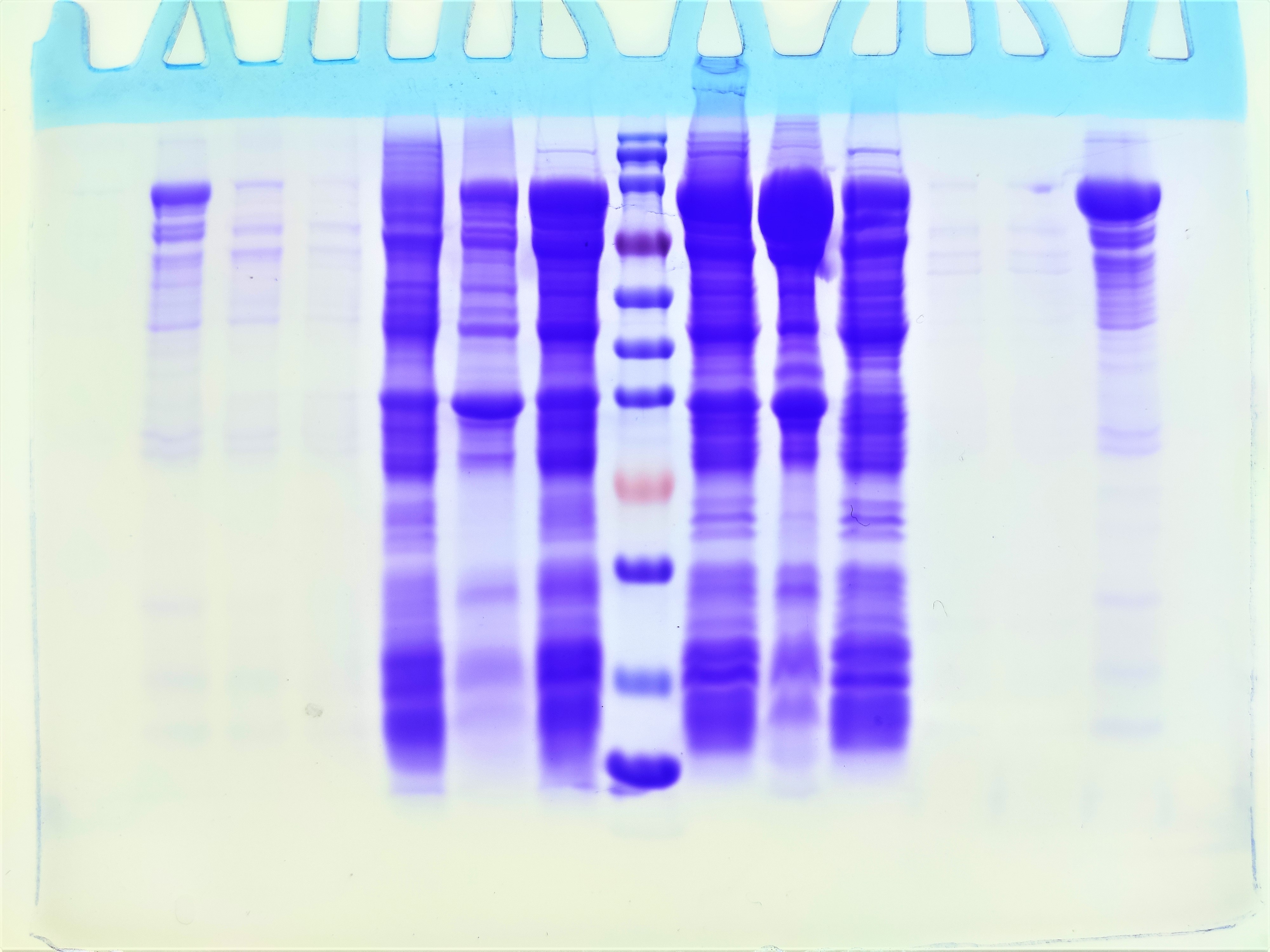

Supplement: Figure 6—figure supplement 3—source data 1. [file elife-101125-fig6-figsupp3-data1.zip › Figure 7-figure supplement 3-source data 1/Figure 7-figure supplement 3-source data 1A-TseP-TseP_E663A.jpg]

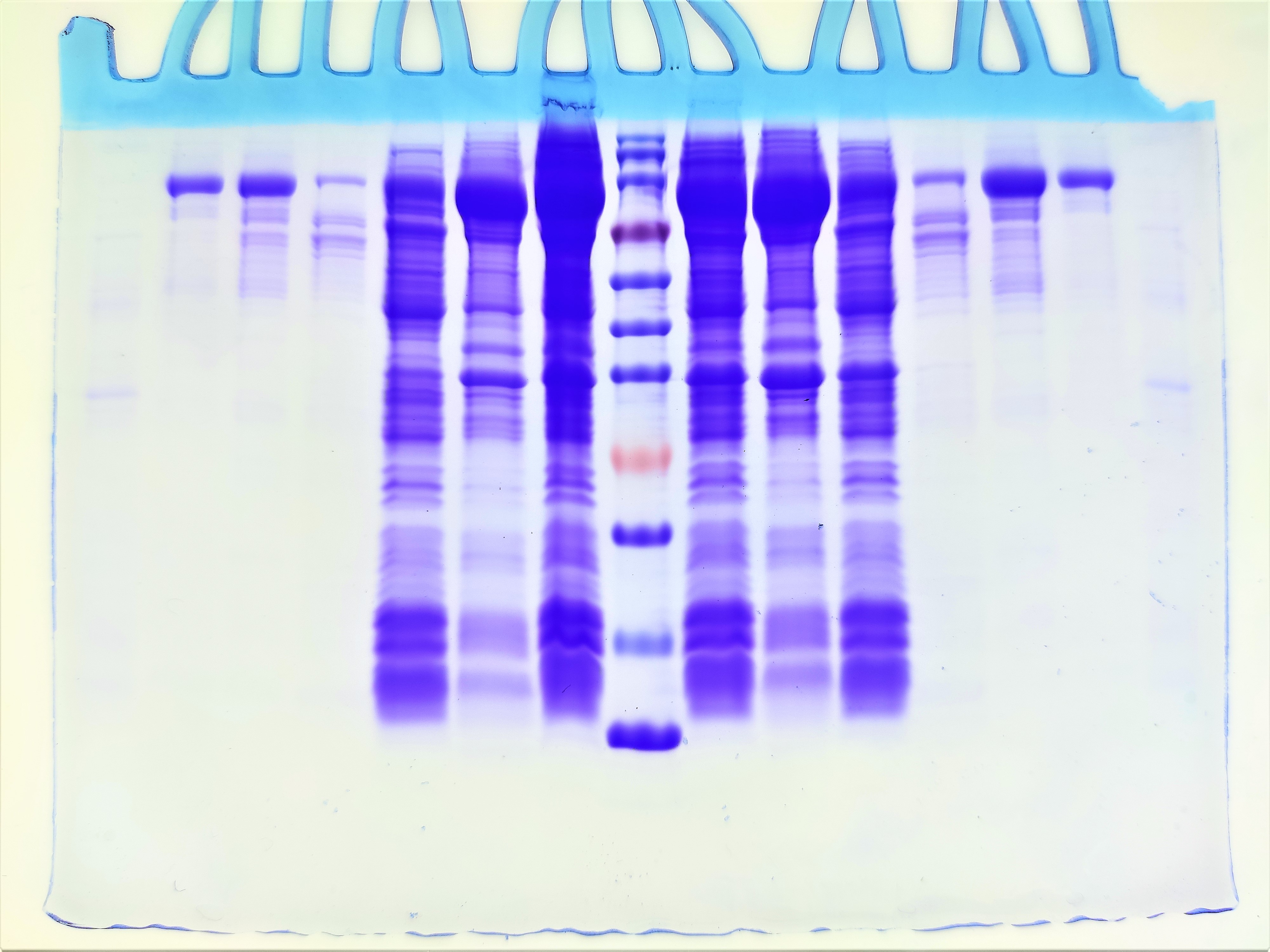

Supplement: Figure 6—figure supplement 3—source data 1. [file elife-101125-fig6-figsupp3-data1.zip › Figure 7-figure supplement 3-source data 1/Figure 7-figure supplement 3-source data 1B-TseP.jpg]

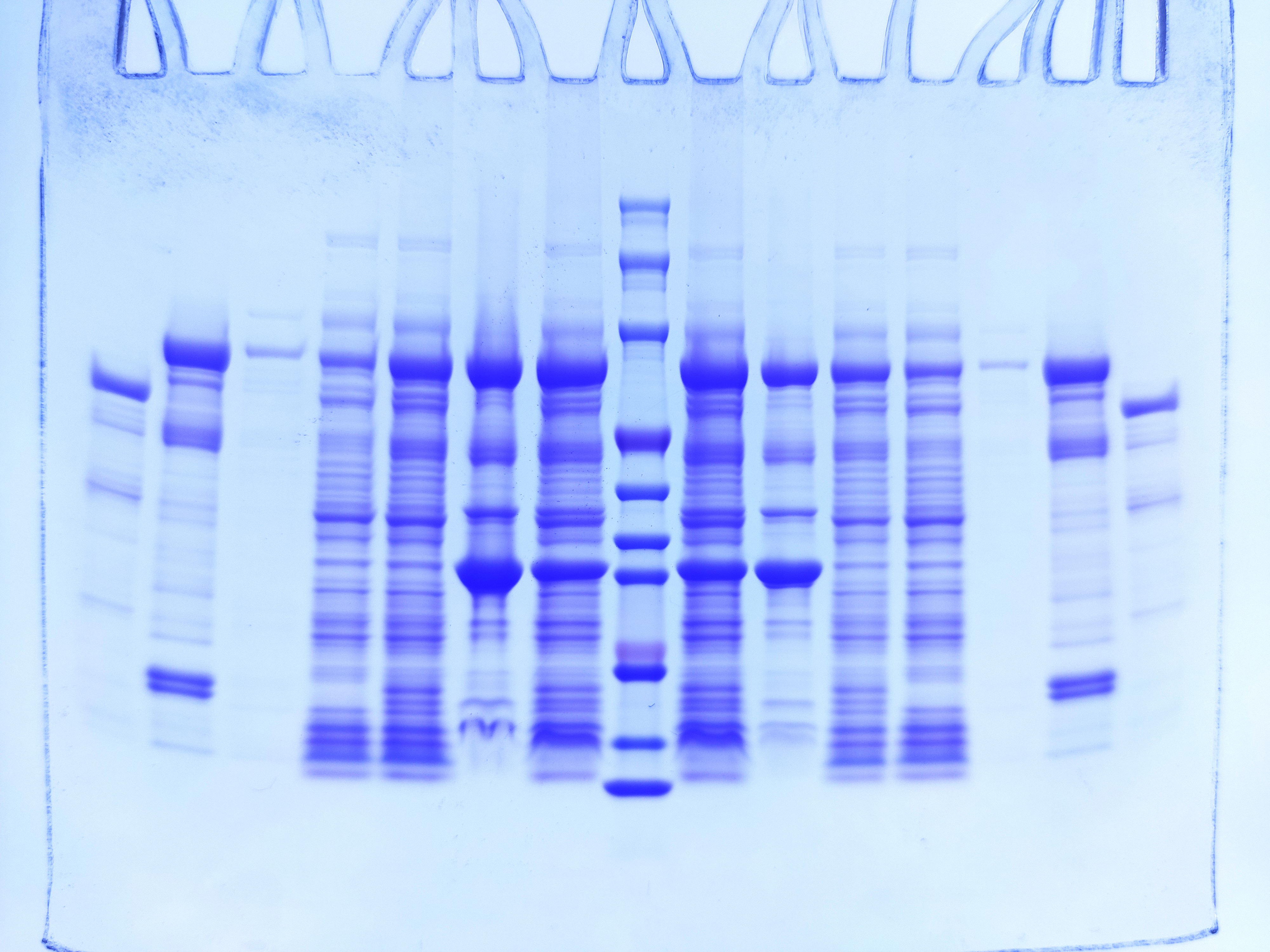

Supplement: Figure 6—figure supplement 3—source data 1. [file elife-101125-fig6-figsupp3-data1.zip › Figure 7-figure supplement 3-source data 1/Figure 7-figure supplement 3-source data 1C-TsePN_H109A-TsePN_H339A.jpg]

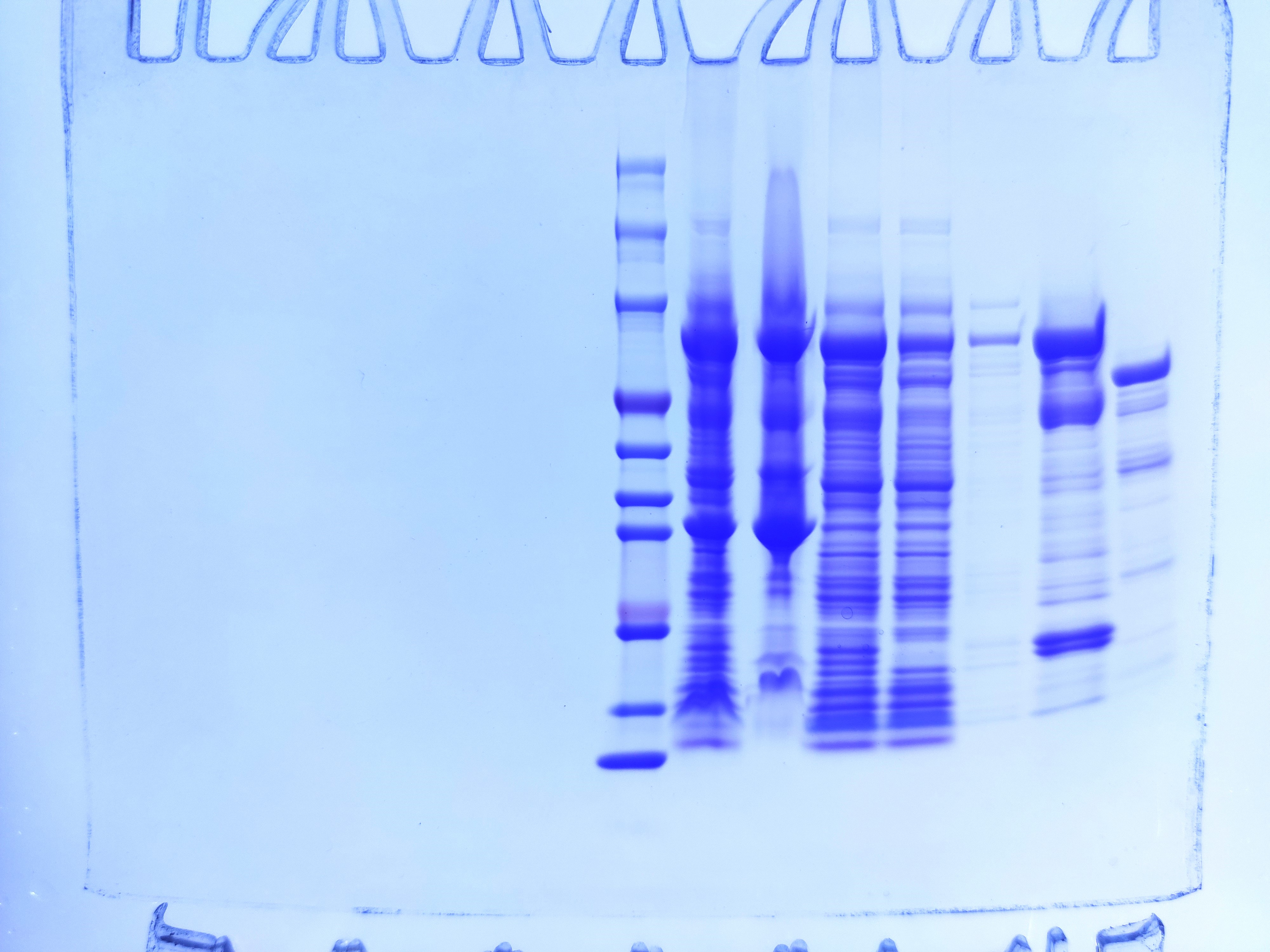

Supplement: Figure 6—figure supplement 3—source data 1. [file elife-101125-fig6-figsupp3-data1.zip › Figure 7-figure supplement 3-source data 1/Figure 7-figure supplement 3-source data 1C-TsePN_H23A.jpg]

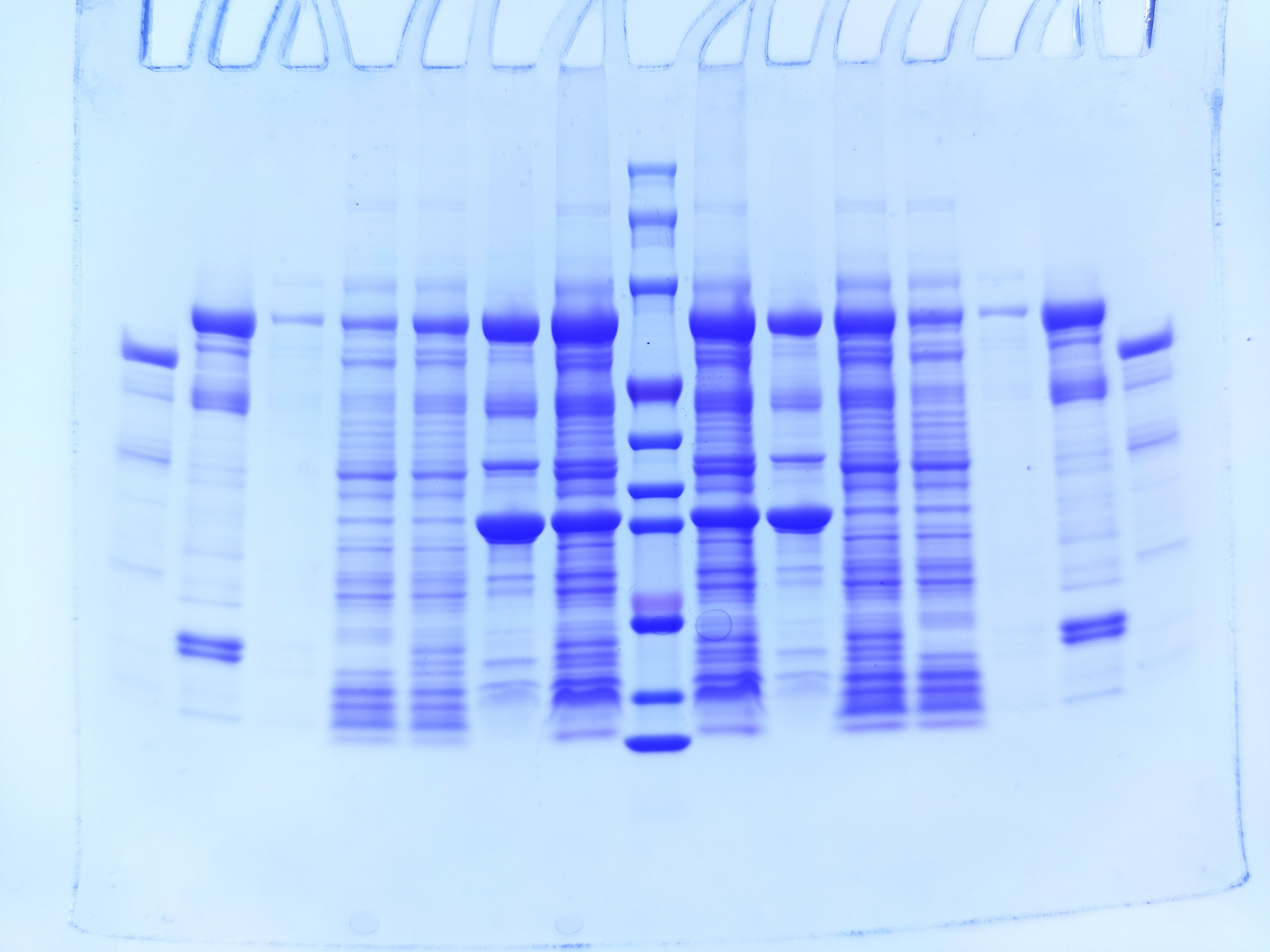

Supplement: Figure 6—figure supplement 3—source data 1. [file elife-101125-fig6-figsupp3-data1.zip › Figure 7-figure supplement 3-source data 1/Figure 7-figure supplement 3-source data 1C-TsePN_H359A-TsePN_E539A.jpg]

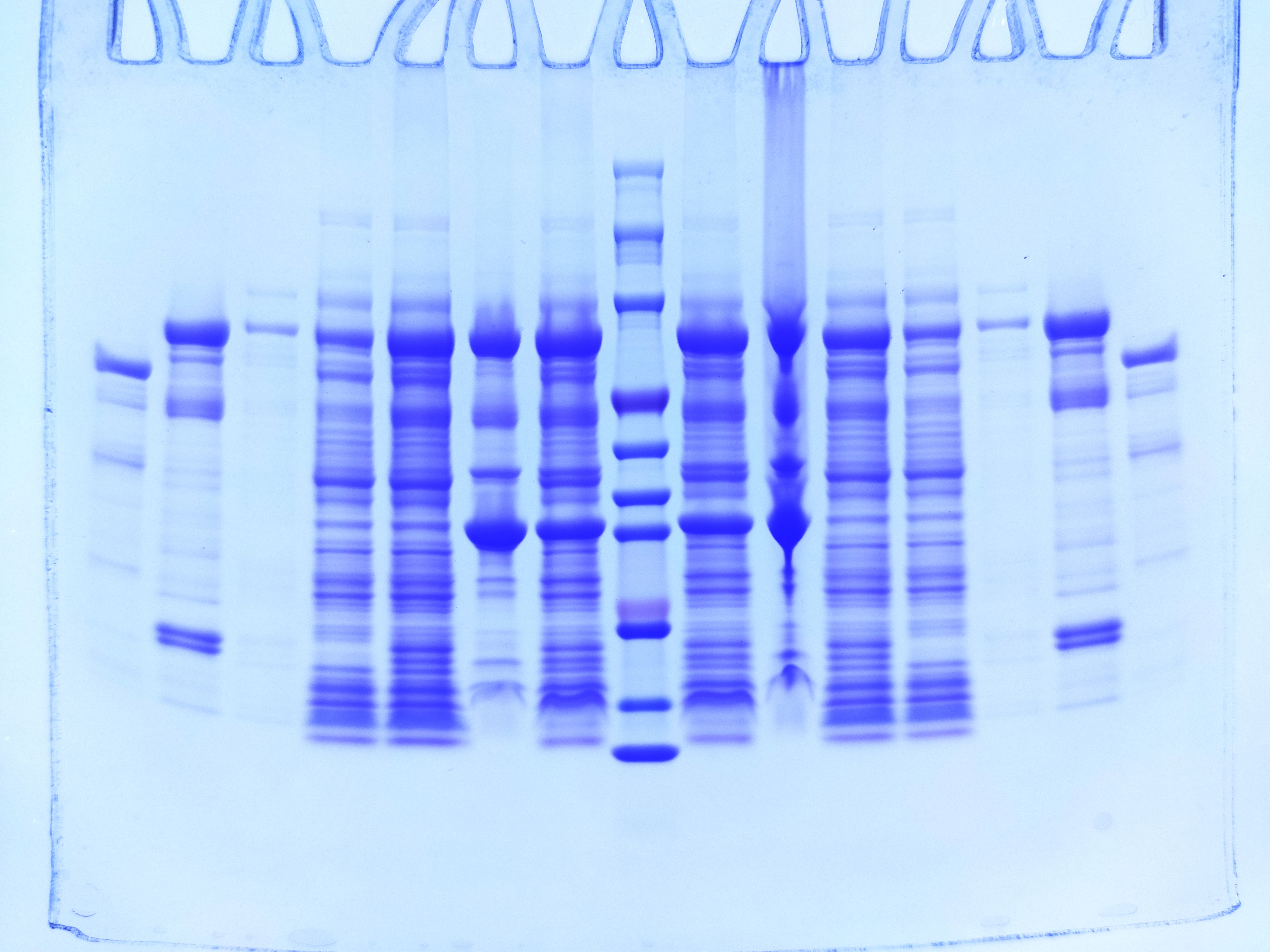

Supplement: Figure 6—figure supplement 3—source data 1. [file elife-101125-fig6-figsupp3-data1.zip › Figure 7-figure supplement 3-source data 1/Figure 7-figure supplement 3-source data 1C-TsePN-TsePN_H19A.jpg]

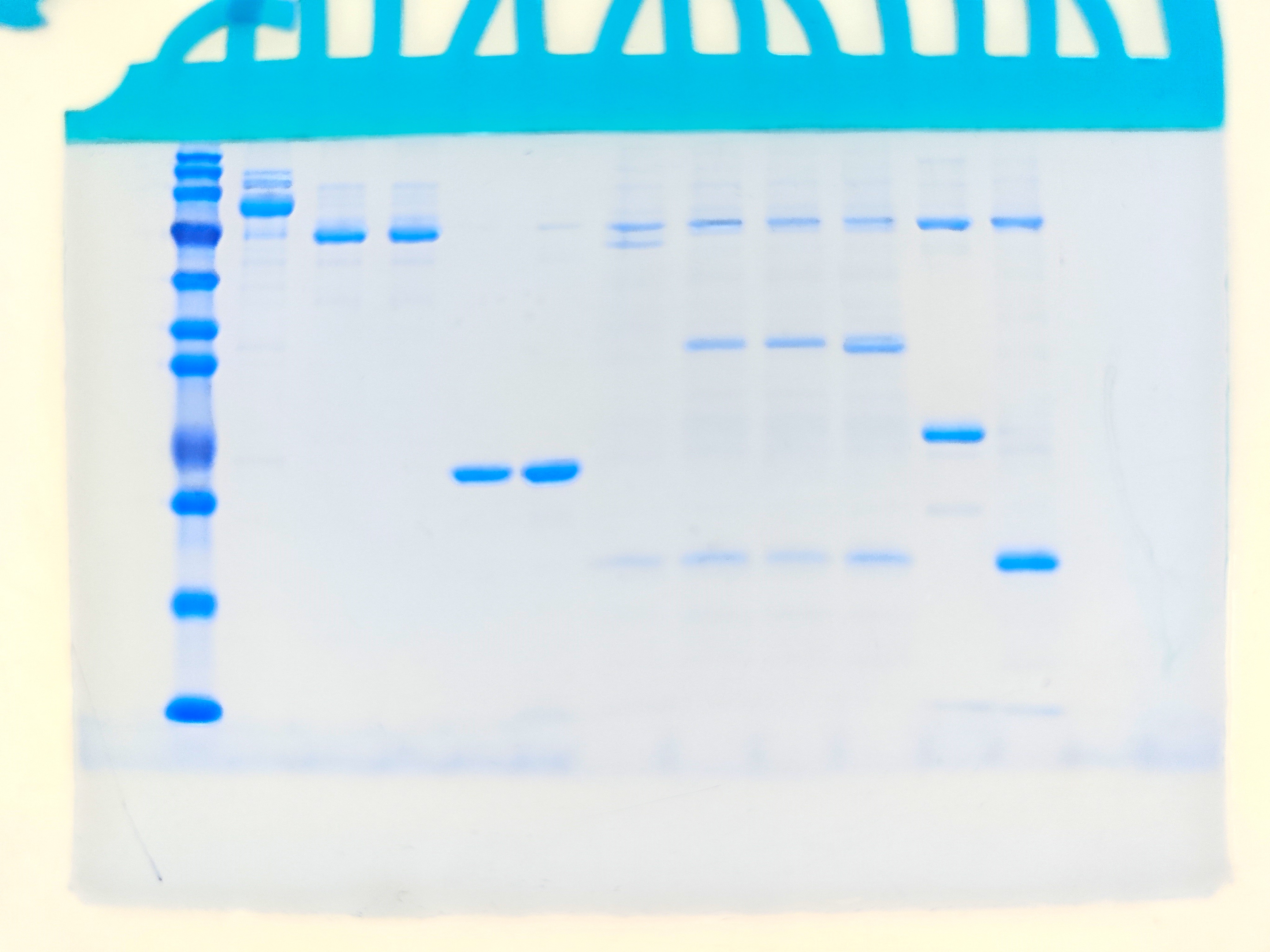

Supplement: Figure 6—figure supplement 3—source data 1. [file elife-101125-fig6-figsupp3-data1.zip › Figure 7-figure supplement 3-source data 1/Figure 7-figure supplement 3-source data 1D-Homolog-proteins.jpg]

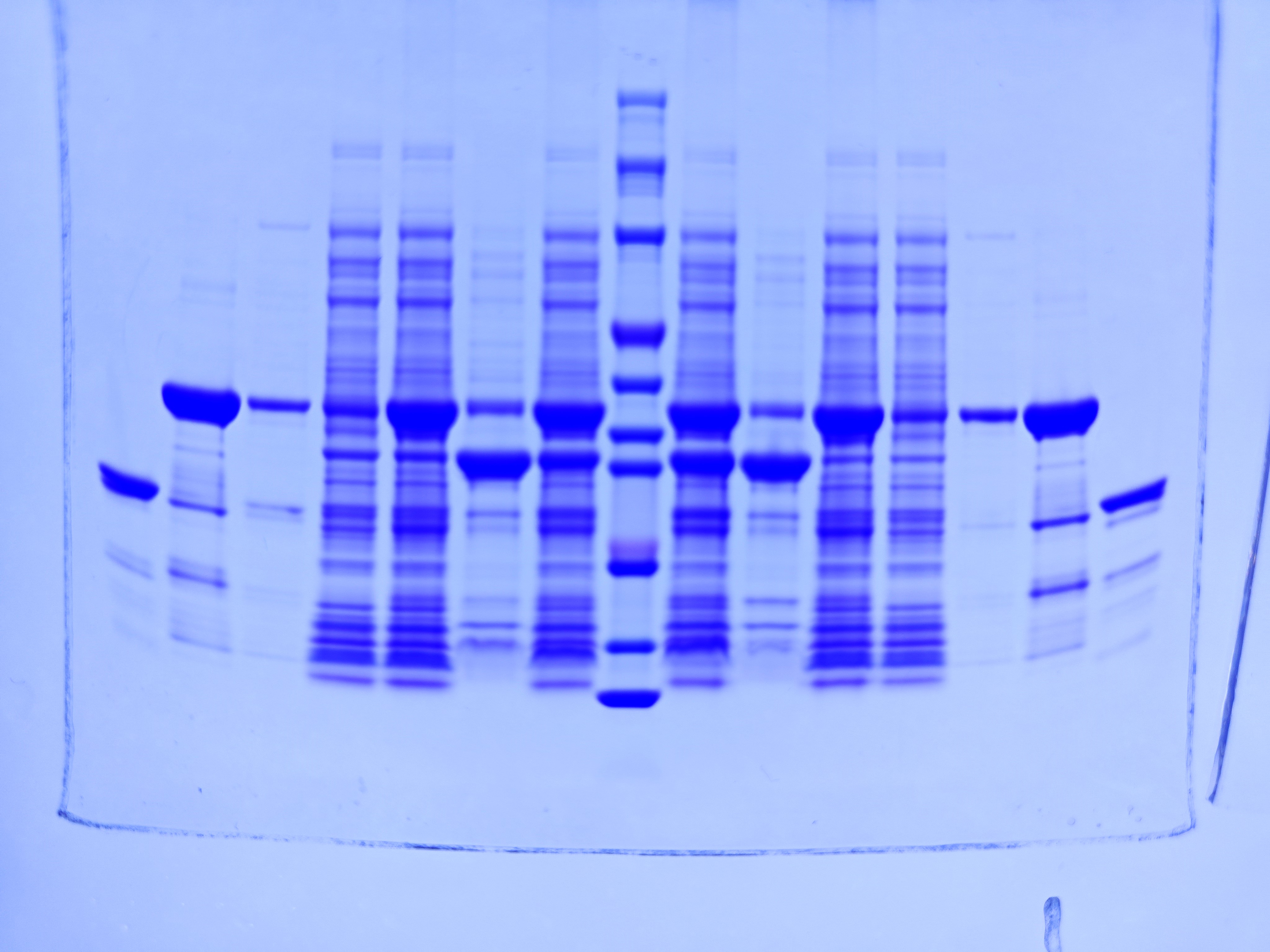

Supplement: Figure 6—figure supplement 3—source data 1. [file elife-101125-fig6-figsupp3-data1.zip › Figure 7-figure supplement 3-source data 1/Figure 7-figure supplement 3-source data 1E-TsePC-TsePC_E663D.jpg]

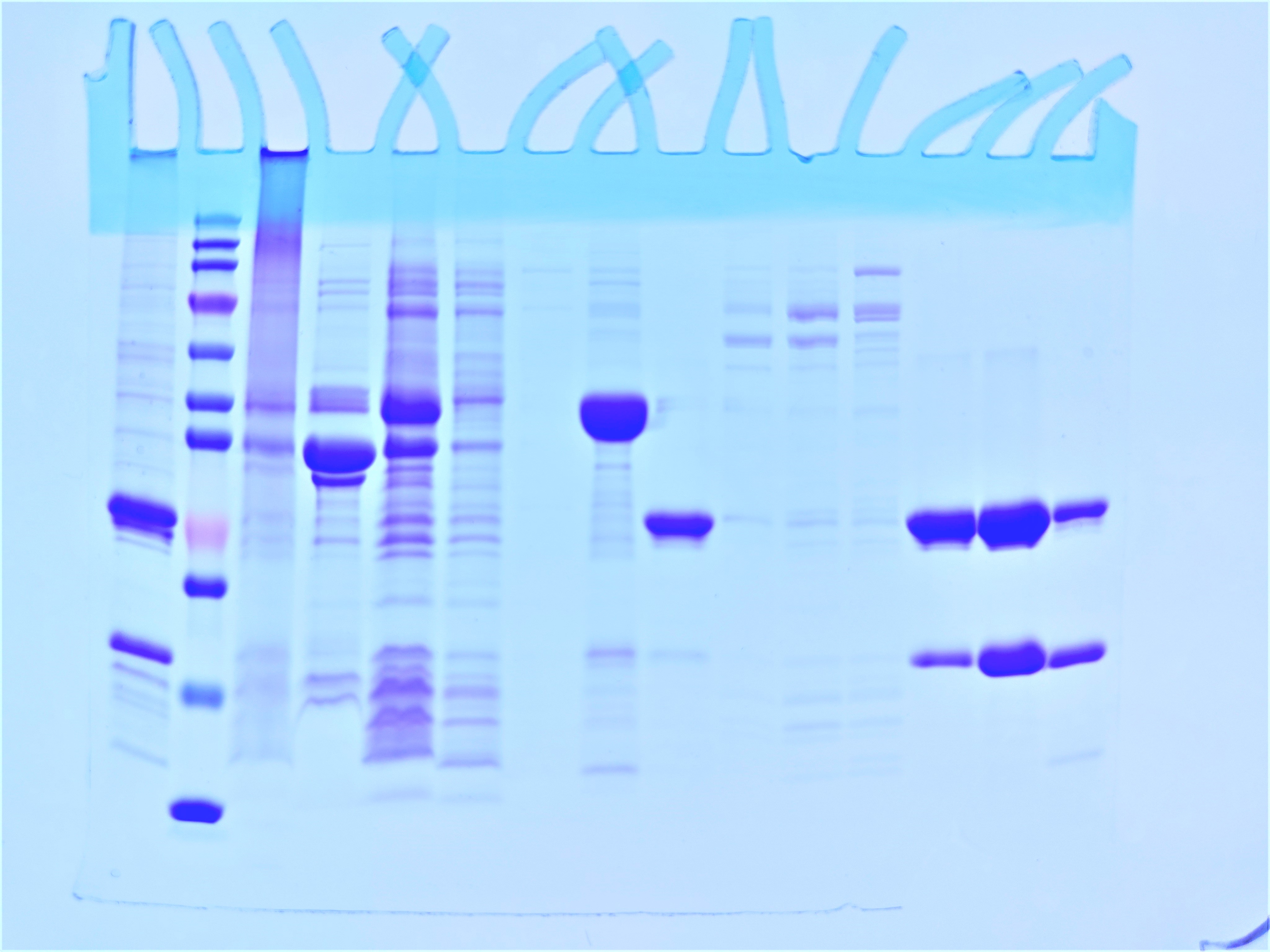

Supplement: Figure 6—figure supplement 3—source data 1. [file elife-101125-fig6-figsupp3-data1.zip › Figure 7-figure supplement 3-source data 1/Figure 7-figure supplement 3-source data 1F-TsePC_4+.jpg]

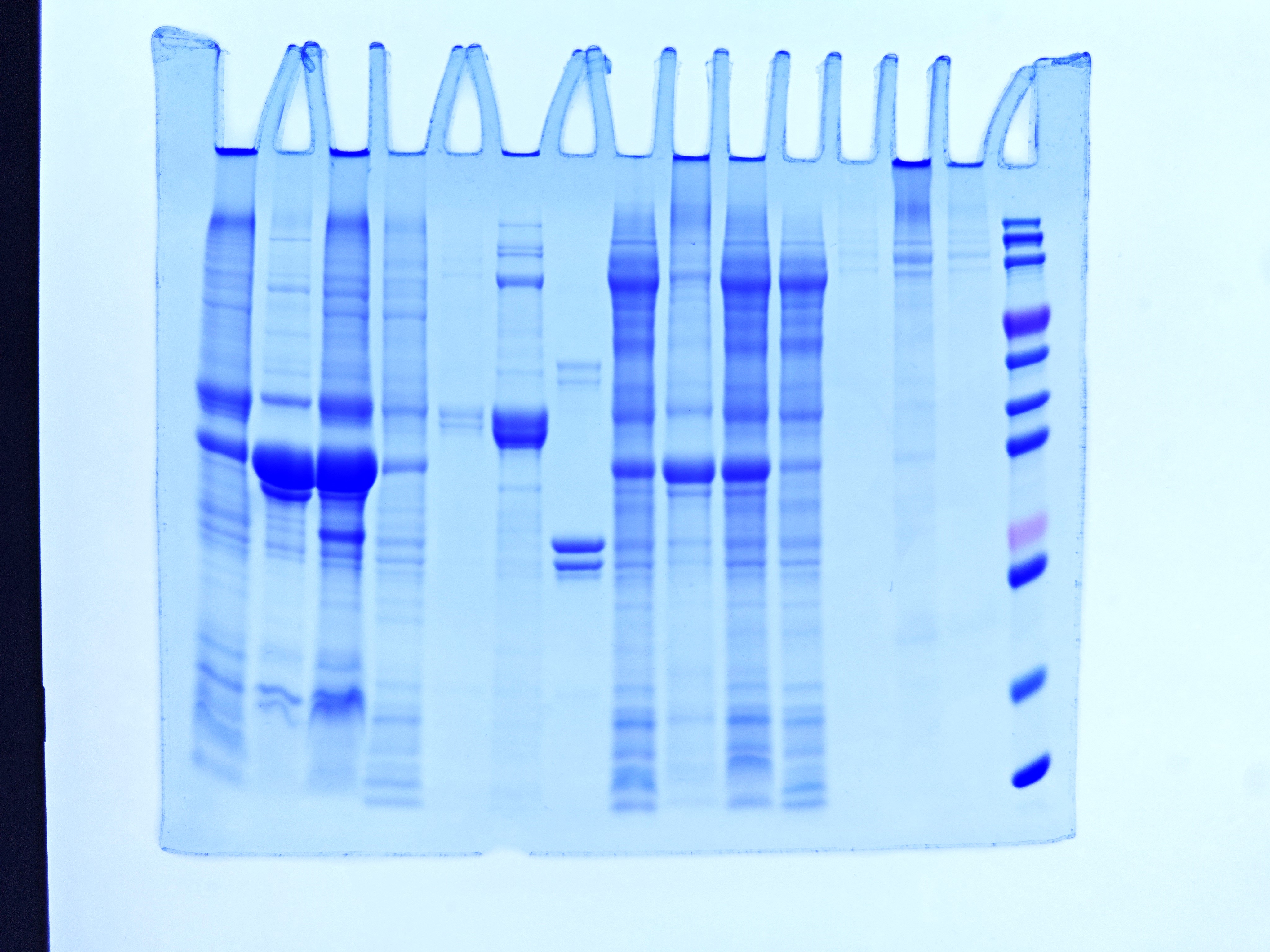

Supplement: Figure 6—figure supplement 3—source data 1. [file elife-101125-fig6-figsupp3-data1.zip › Figure 7-figure supplement 3-source data 1/Figure 7-figure supplement 3-source data 1F-TsePC_D841K_E845K.jpg]

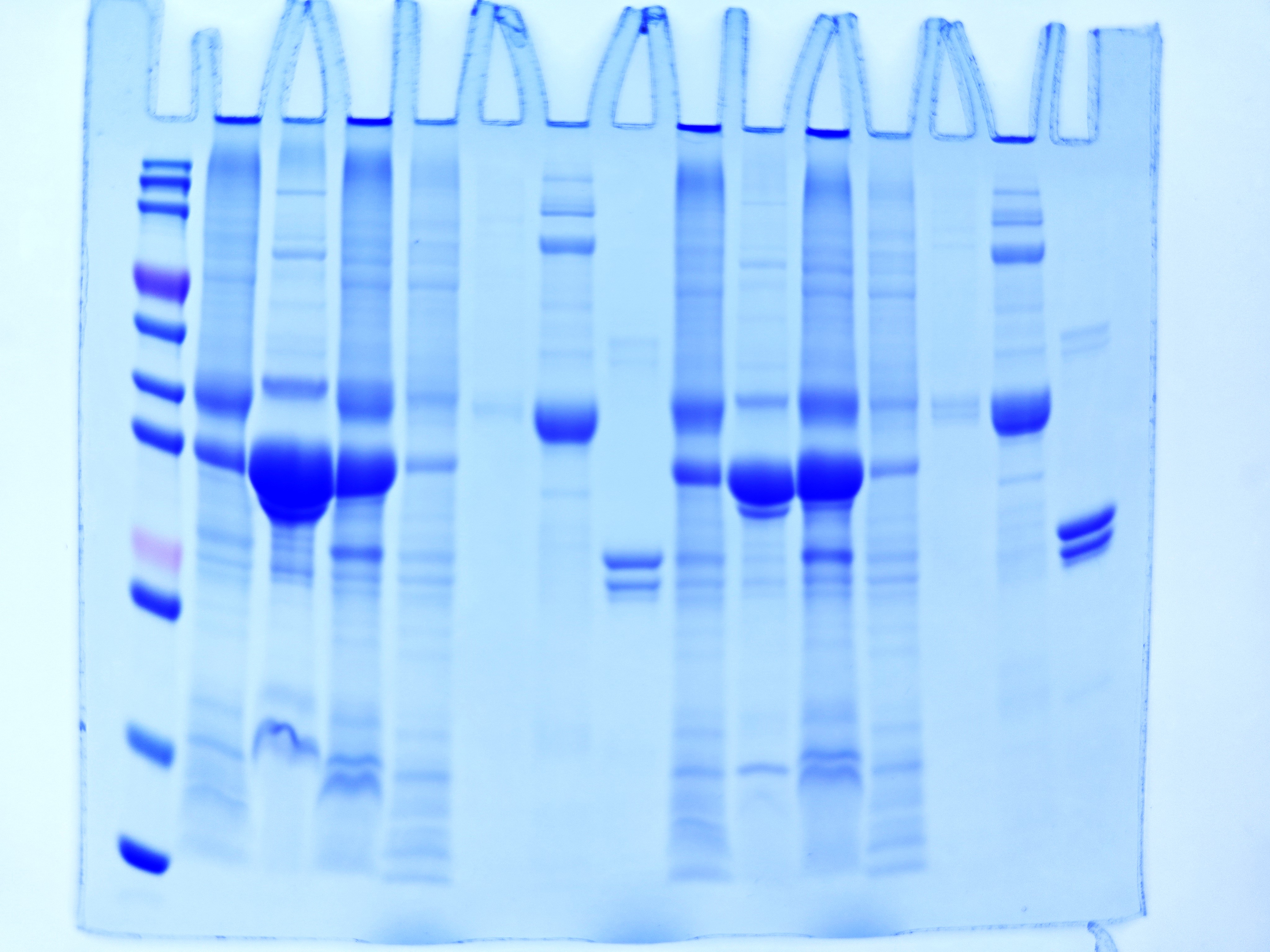

Supplement: Figure 6—figure supplement 3—source data 1. [file elife-101125-fig6-figsupp3-data1.zip › Figure 7-figure supplement 3-source data 1/Figure 7-figure supplement 3-source data 1F-TsePC_E604K-TsePC_D841K.jpg]
