# Supplementary material for: Amidase and lysozyme dual functions in TseP reveal a new family of chimeric effectors in the type VI secretion system
Source: eLife. 2025 Mar 10;13:RP101125. doi: 10.7554/eLife.101125 (PMC11893102; doi:10.7554/eLife.101125)
Supplement: Figure 1—source data 2. [file elife-101125-fig1-data2.zip › Figure 1-source data 2/Figure 1-source data 2.pdf]

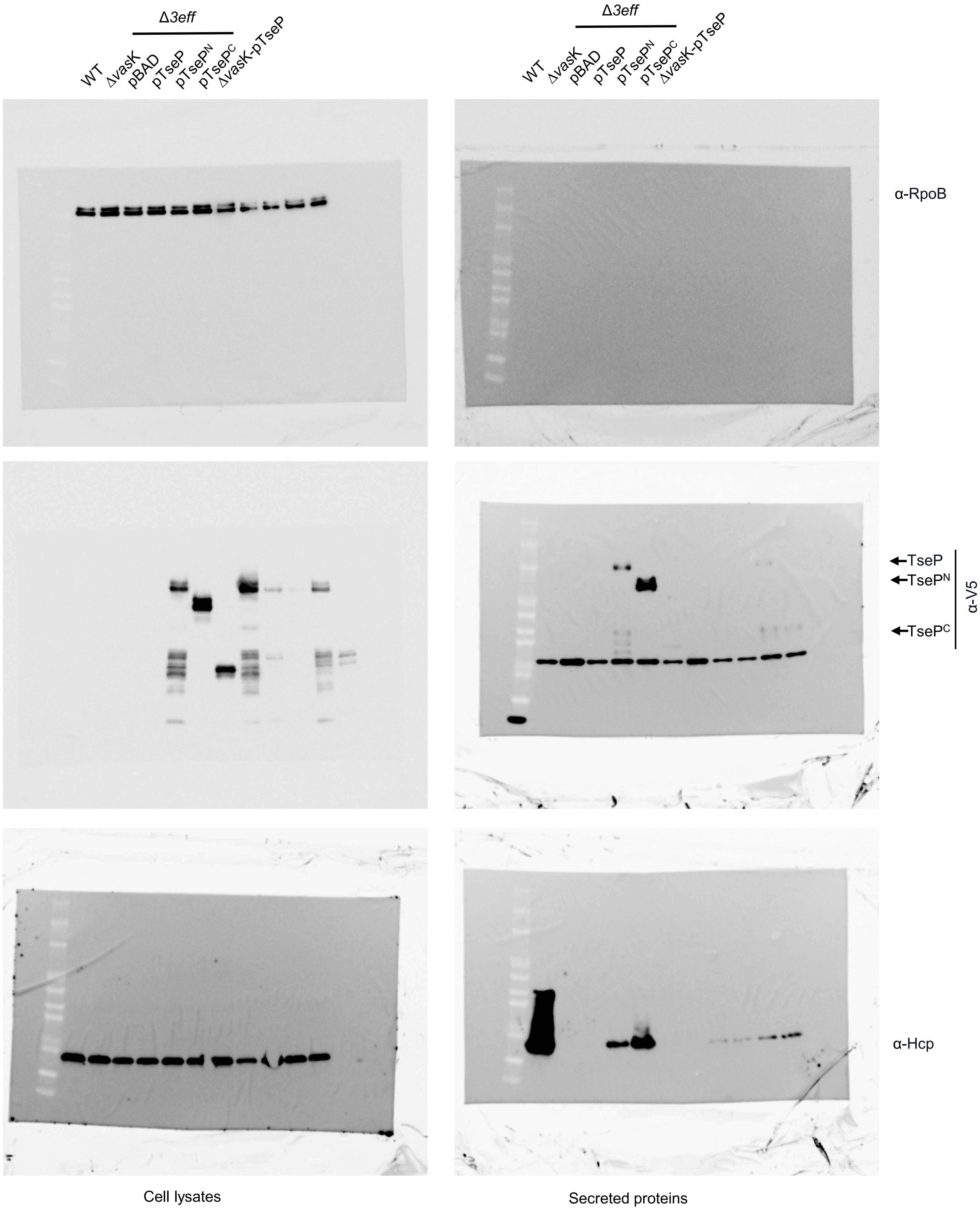

Figure 1A, Secretion analysis of TseP, TseP^N, and TseP^C in the SSU triple effector deletion mutant (Δ*3eff*). A schematic of the TseP N-terminus (TseP^N, 1-603 aa) and C-terminus (TseP^C, 604-845 aa) is depicted at the top. Hcp serves as a positive control for T6SS secretion. Hcp, RpoB, and 3V5-tagged TseP proteins were detected using specific antibodies.

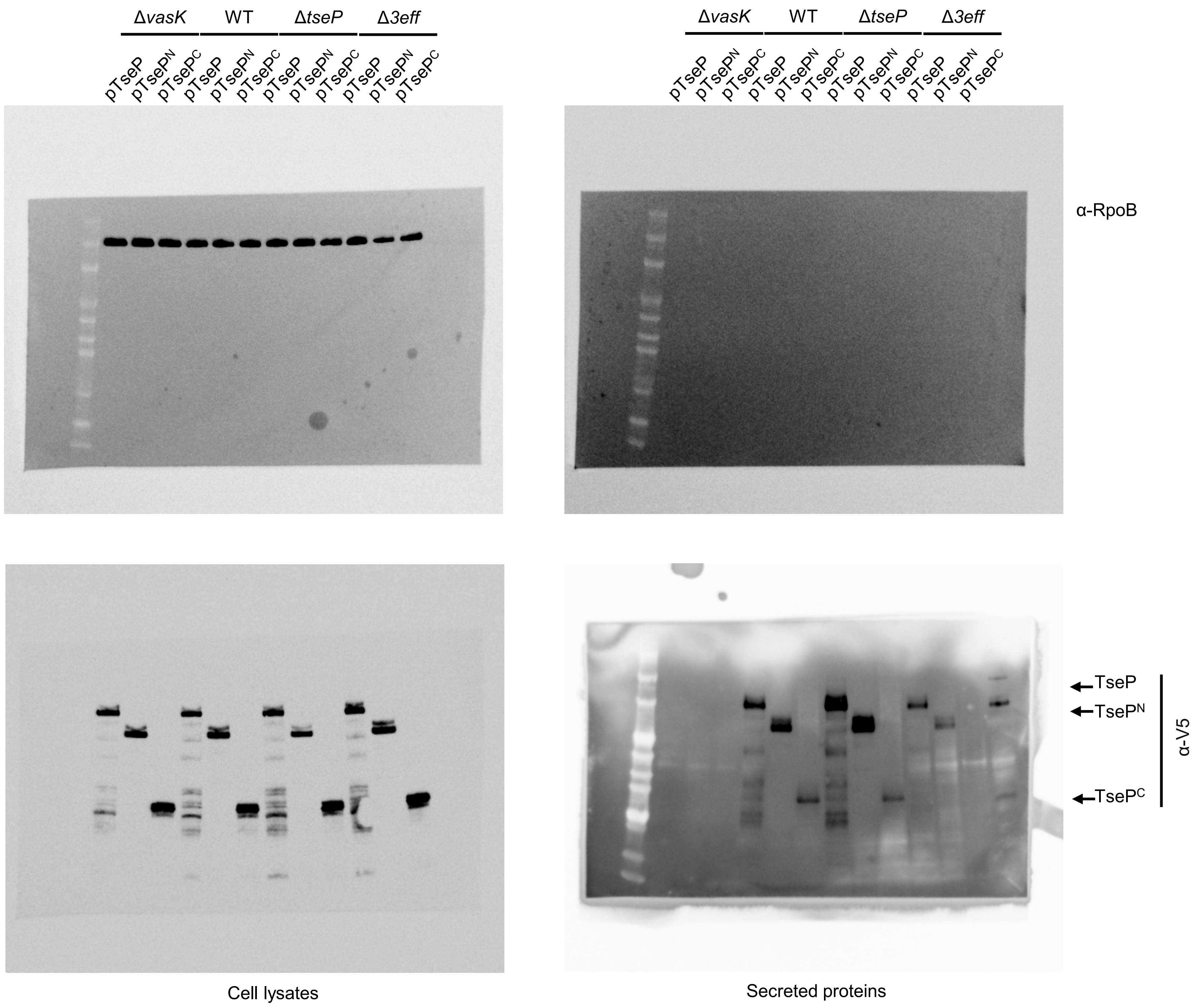

Figure 1 D, Secretion analysis of TseP, TseP^{N}, and TseP^{C} in SSU wild type, Δ*vasK*, Δ*tseP*, and Δ*3eff* mutants. TseP, TseP^{N}, and TseP^{C} were tagged with a 3V5 C-terminal tag and expressed on pBAD vectors. RpoB serves as an equal loading and autolysis control.

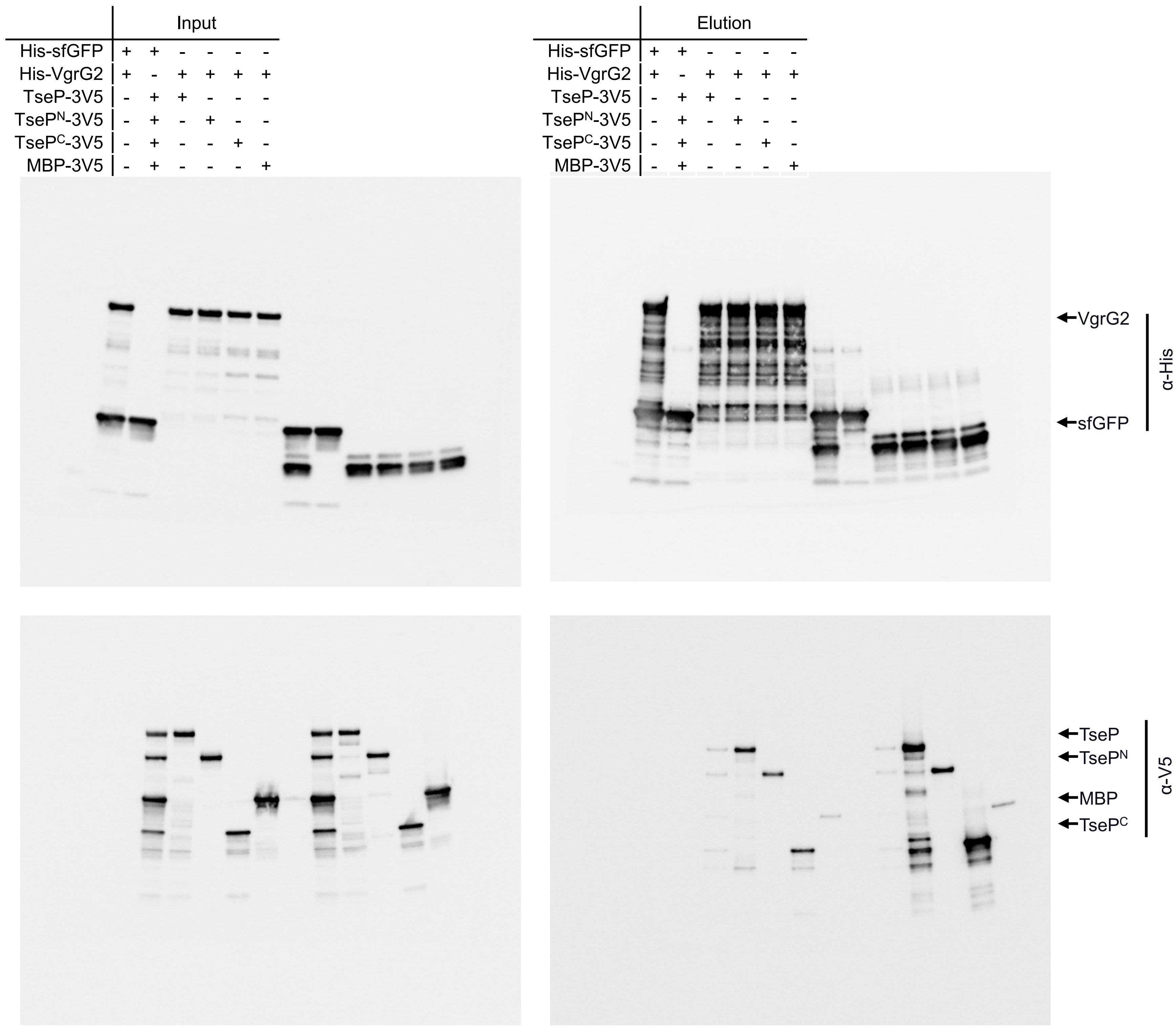

Figure 1E, Pull-down analysis of VgrG2 with TseP, $TseP^N$, and $TseP^C$. His-tagged VgrG2 and 3V5-tagged TseP, $TseP^N$, or $TseP^C$ were used. His-tagged sfGFP and 3V5-tagged MBP were used as controls.
