# Supplementary material for: Amidase and lysozyme dual functions in TseP reveal a new family of chimeric effectors in the type VI secretion system
Source: eLife. 2025 Mar 10;13:RP101125. doi: 10.7554/eLife.101125 (PMC11893102; doi:10.7554/eLife.101125)
Supplement: Figure 1—figure supplement 1—source data 2. [file elife-101125-fig1-figsupp1-data2.zip › Figure 1-figure supplement 1-source data 2/Figure 1-figure supplement 1-source data 2.pdf]

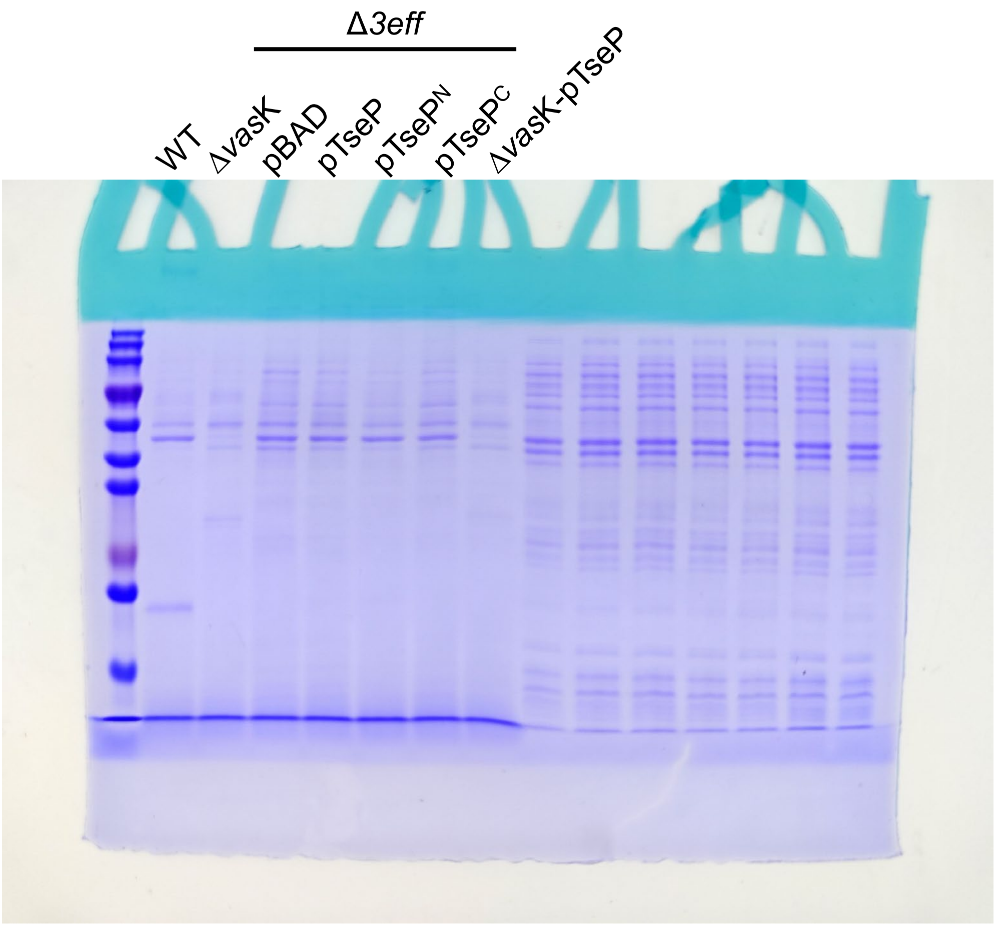

Figure 1-figure supplement 1. Staining SDS-PAGE of secretion of TseP, TseP^{N}, and TseP^{C} in the SSU triple effector deletion mutant (Δ*3eff*). The secretion proteins were visualized by SDS-PAGE and stained with Coomassie brilliant blue dye.
