# Supplementary material for: Amidase and lysozyme dual functions in TseP reveal a new family of chimeric effectors in the type VI secretion system
Source: eLife. 2025 Mar 10;13:RP101125. doi: 10.7554/eLife.101125 (PMC11893102; doi:10.7554/eLife.101125)
Supplement: Figure 2—source data 2. [file elife-101125-fig2-data2.zip › Figure 2-source data 2/Figure 2-source data 2.pdf]

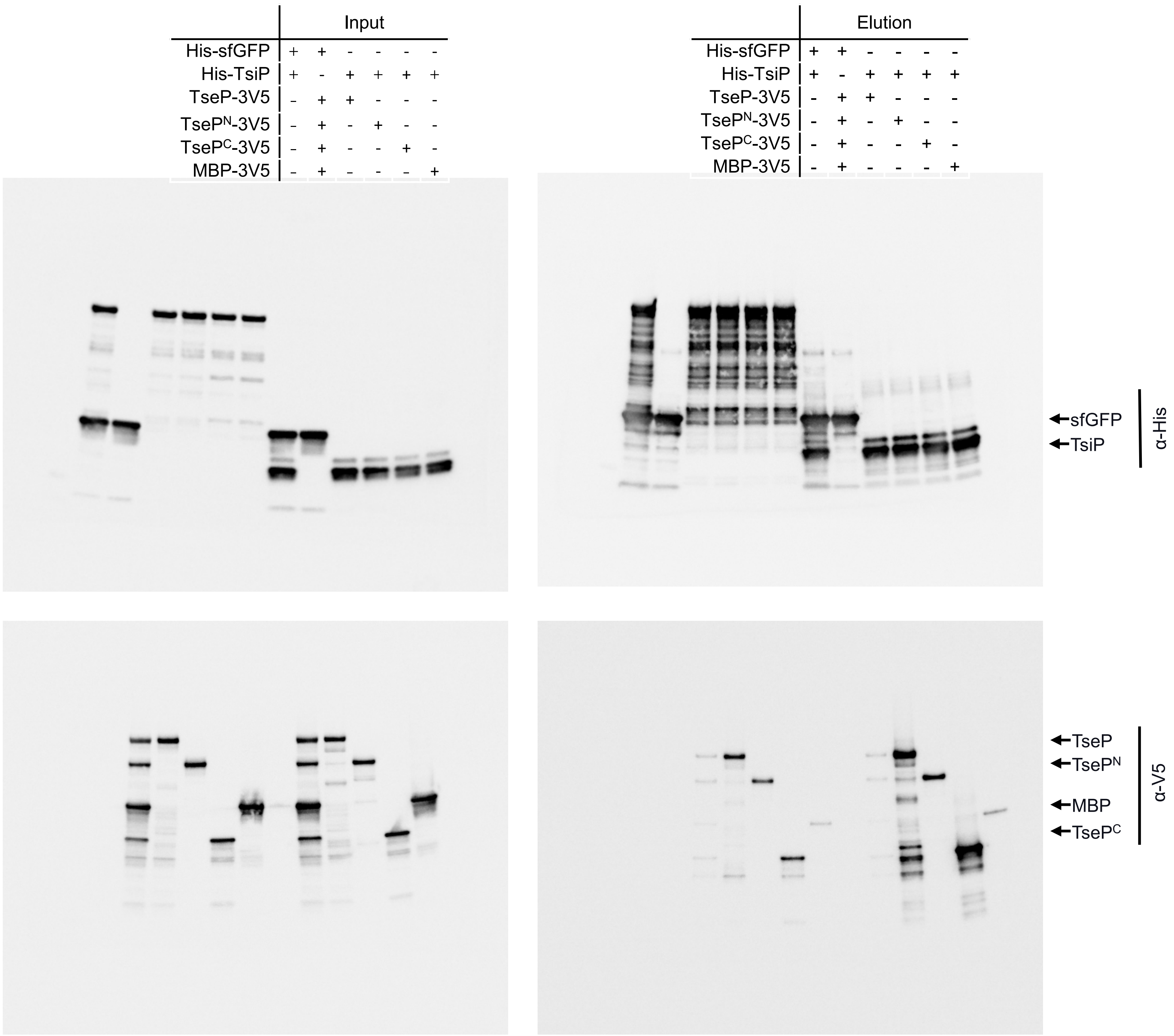

Figure 2G, Pull-down analysis of TsiP with TseP, TsePN, and TsePC. His-tagged TsiP and 3V5-tagged TseP, TsePN, or TsePC were used. His-tagged sfGFP and 3V5-tagged MBP were used as controls.
