# Supplementary material for: Amidase and lysozyme dual functions in TseP reveal a new family of chimeric effectors in the type VI secretion system
Source: eLife. 2025 Mar 10;13:RP101125. doi: 10.7554/eLife.101125 (PMC11893102; doi:10.7554/eLife.101125)
Supplement: Figure 6—source data 2. [file elife-101125-fig6-data2.zip › Figure 6-source data 2/Figure 6-source data 2.pdf]

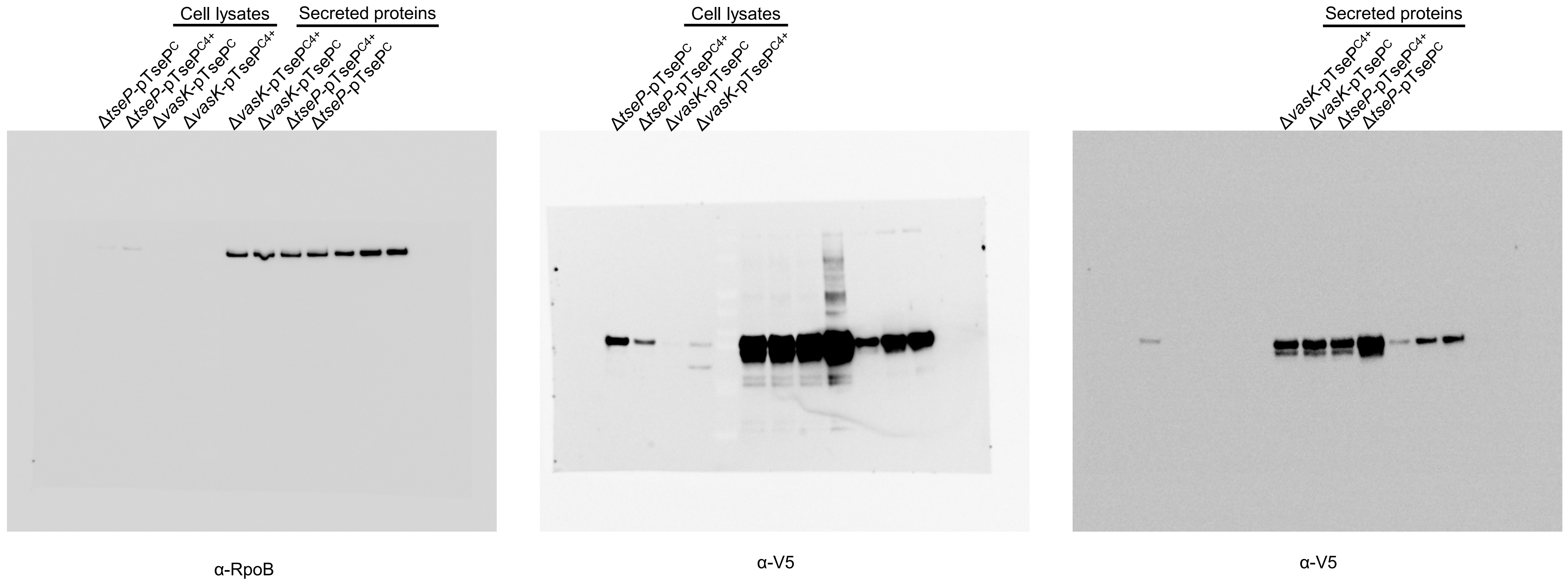

Figure 6E, Secretion analysis of TseP^{C} and TseP^{C4+} in the Δ*tseP* mutant. RpoB serves as an equal loading and autolysis control. RpoB and 3V5-tagged TseP^{C} proteins were detected using specific antibodies.
