# Supplementary material for: Amidase and lysozyme dual functions in TseP reveal a new family of chimeric effectors in the type VI secretion system
Source: eLife. 2025 Mar 10;13:RP101125. doi: 10.7554/eLife.101125 (PMC11893102; doi:10.7554/eLife.101125)
Supplement: Figure 6—figure supplement 2—source data 2. [file elife-101125-fig6-figsupp2-data2.zip › Figure 7-figure supplement 2-source data 2/Figure 7-figure supplement 2-source data 2.pdf]

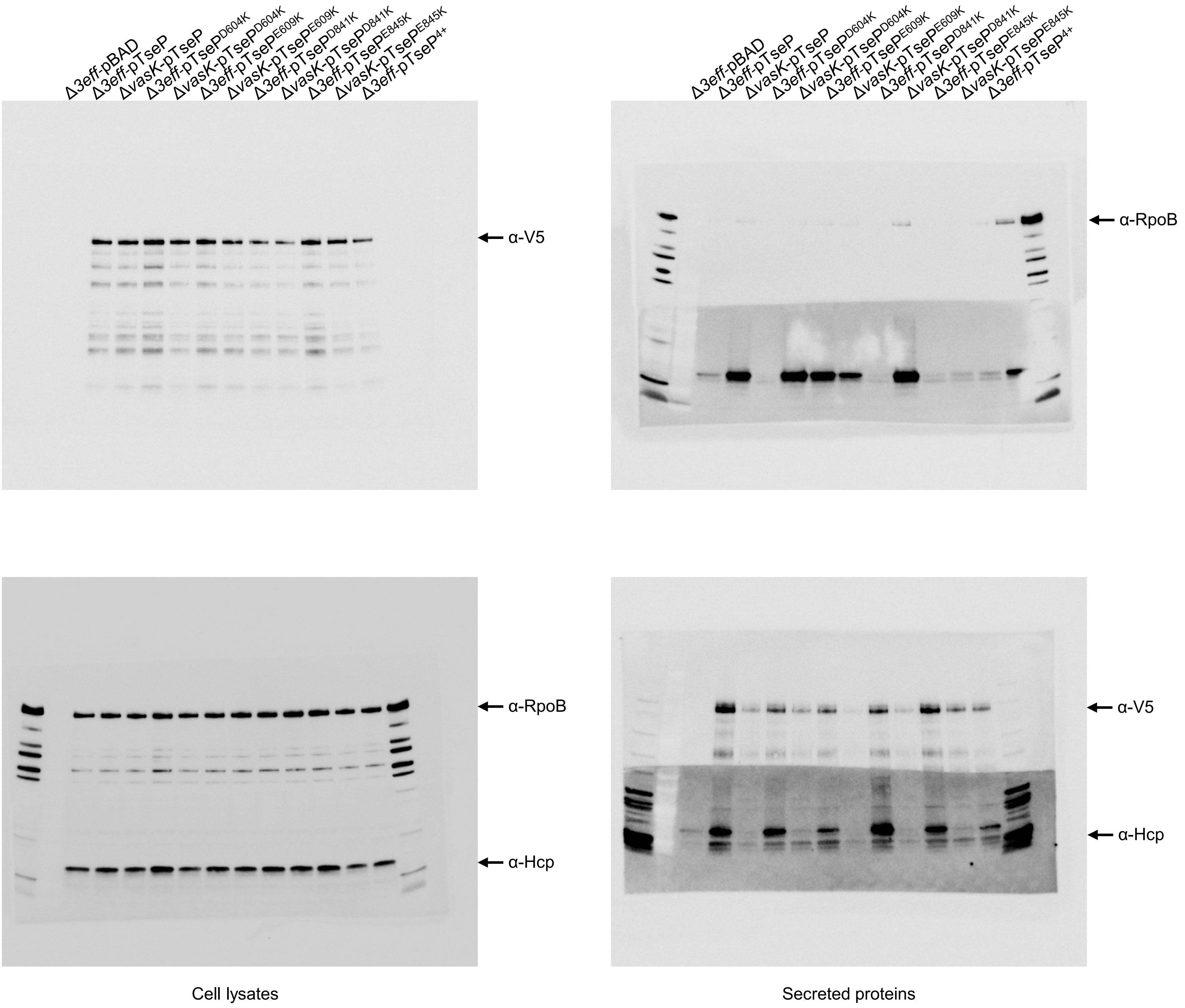

Figure 7-figure supplement 2A, Secretion analysis of Hcp in the Δ*3eff* mutant complemented with different TseP variants. RpoB serves as an equal loading and autolysis control. Hcp, RpoB, and 3V5-tagged TseP proteins were detected using specific antibodies.

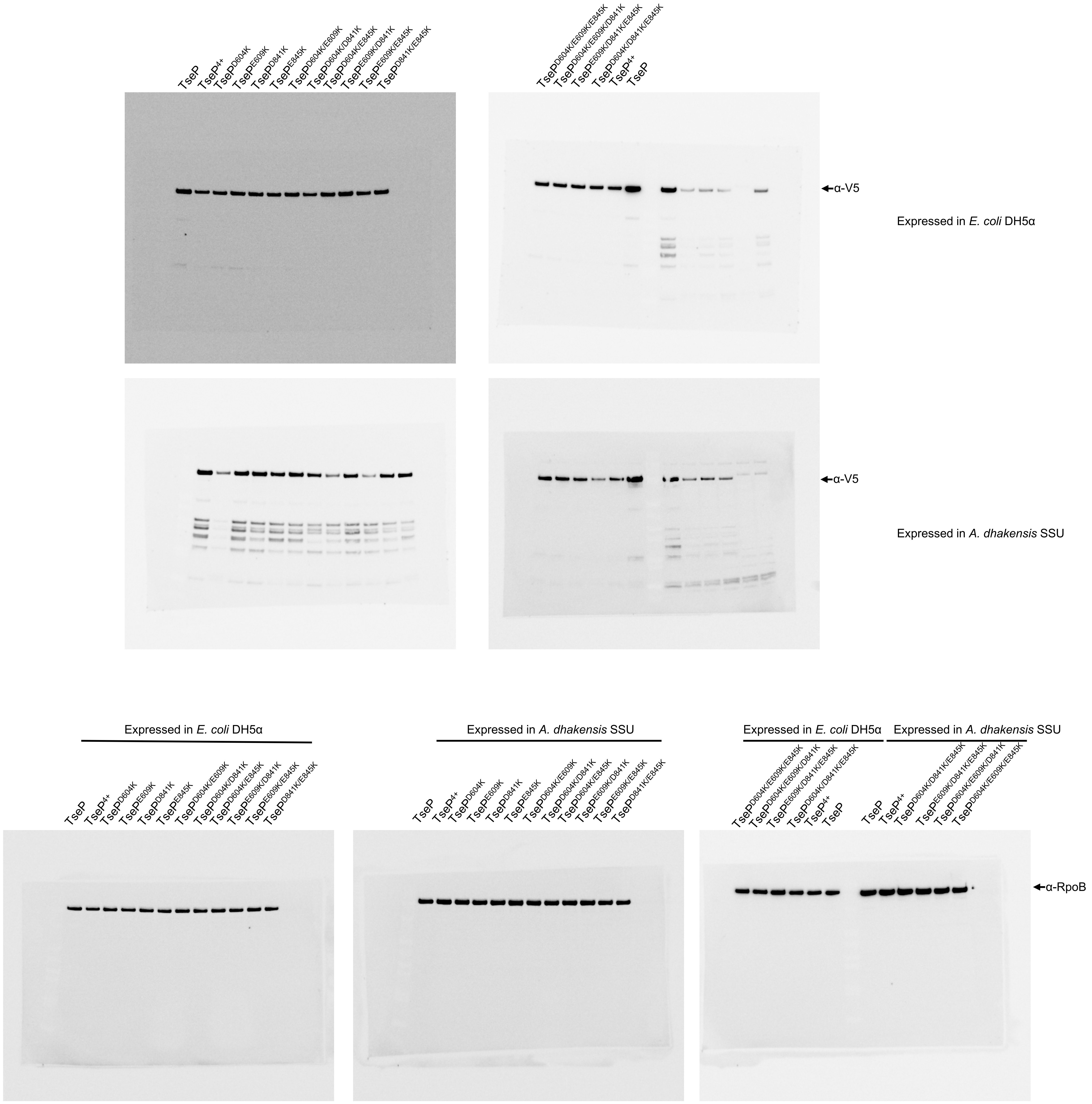

Figure 7-figure supplement 2B, Protein expression of TseP and its variants in *E. coli* and SSU strains. RpoB serves as an equal loading control. RpoB and 3V5-tagged TseP proteins were detected using specific antibodies.
