# Supplementary material for: Amidase and lysozyme dual functions in TseP reveal a new family of chimeric effectors in the type VI secretion system
Source: eLife. 2025 Mar 10;13:RP101125. doi: 10.7554/eLife.101125 (PMC11893102; doi:10.7554/eLife.101125)
Supplement: Figure 6—figure supplement 3—source data 2. [file elife-101125-fig6-figsupp3-data2.zip › Figure 7-figure supplement 3-source data 2/Figure 7-figure supplement 3-source data 2.pdf]

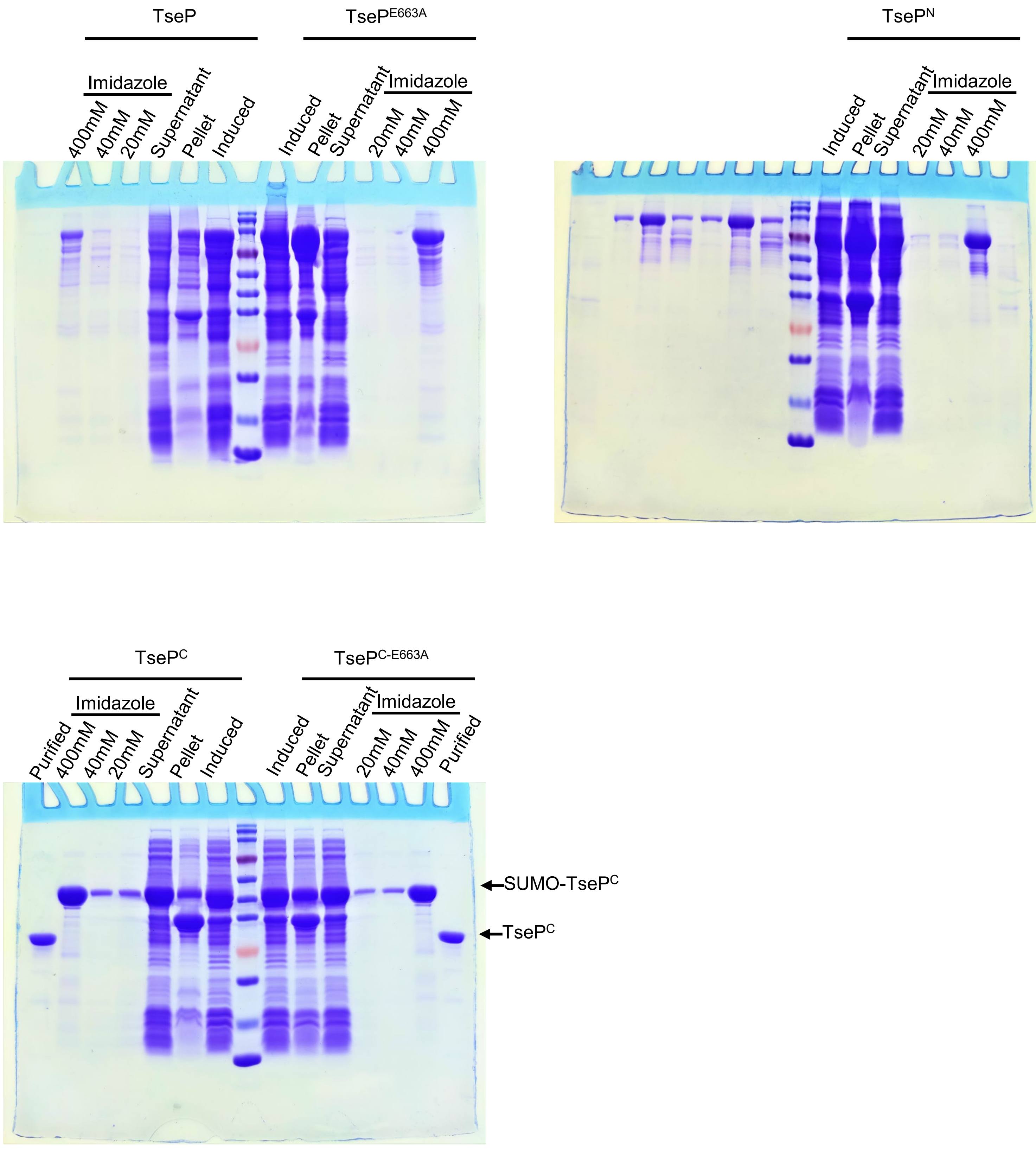

Figure 7-figure supplement 3A, Purification of His-TseP, His-TseP^{E663A}, His-SUMO-TseP^{N}, His-SUMO-TseP^{C}, and His-SUMO-TseP$^{C-E663A}$. The His-SUMO tag was removed by SUMO protease.

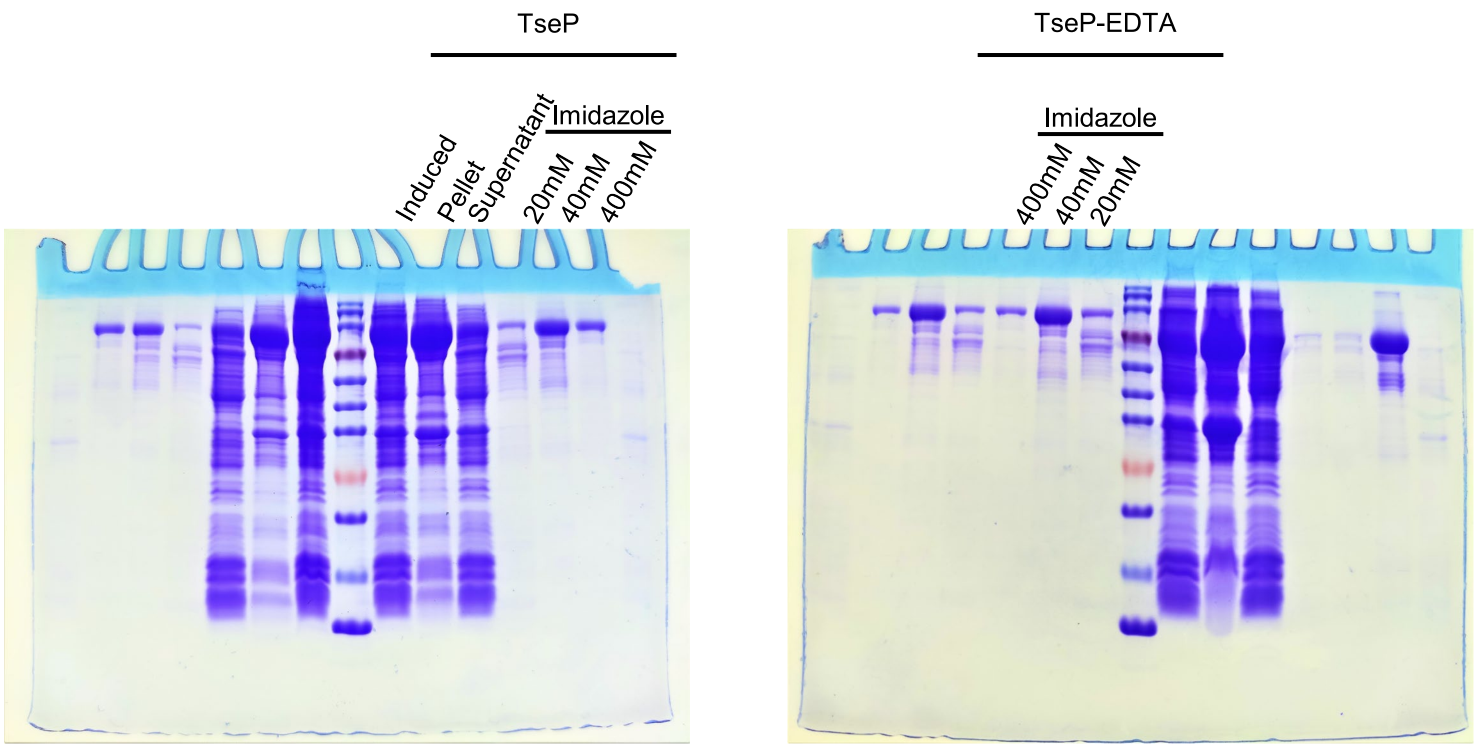

Figure 7-figure supplement 3B, Purification of TseP proteins with or without EDTA.

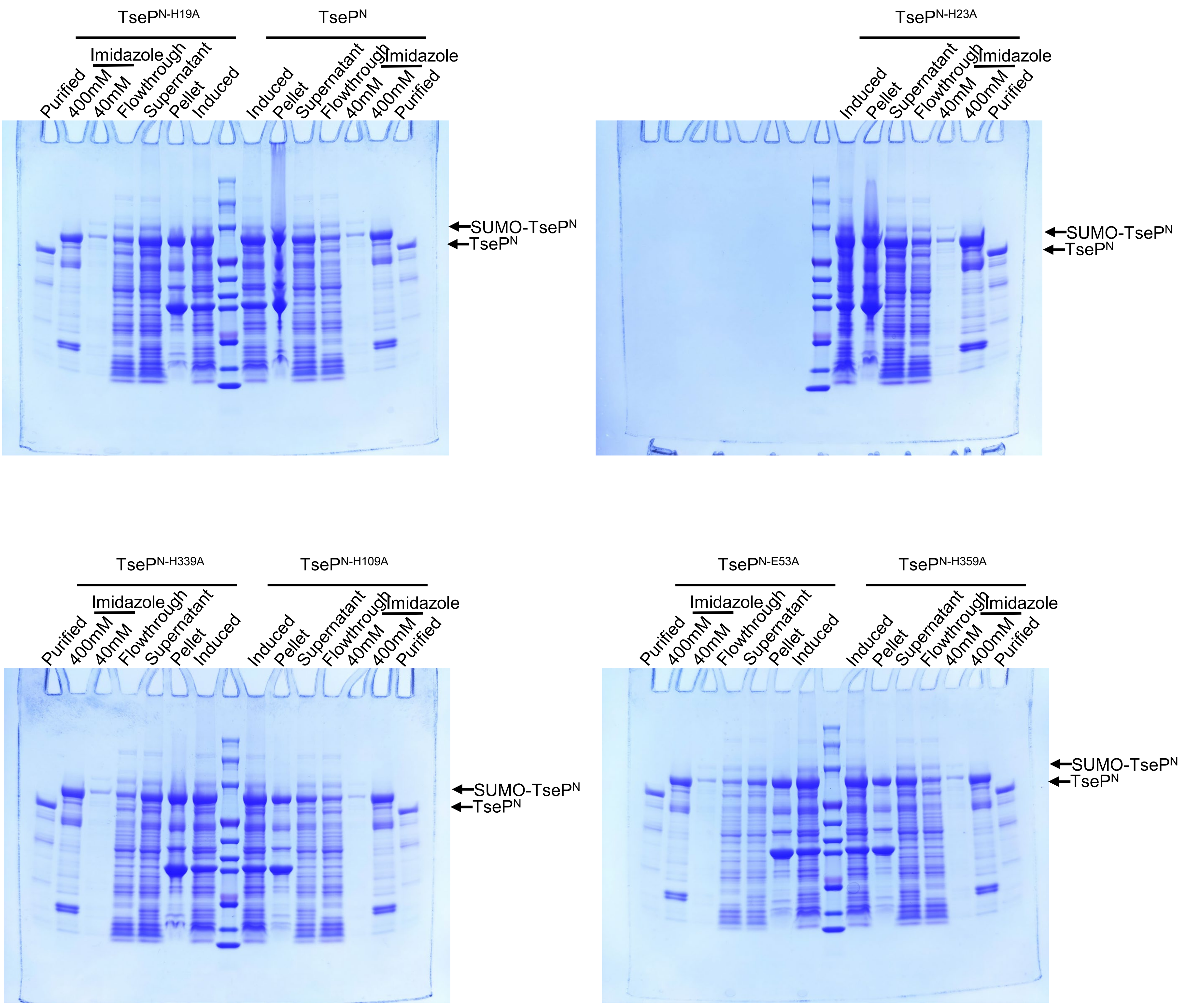

Figure 7-figure supplement 3C, Purification of TseP^{N} variants with a His-SUMO tag. The His-SUMO tag was removed by SUMO protease.

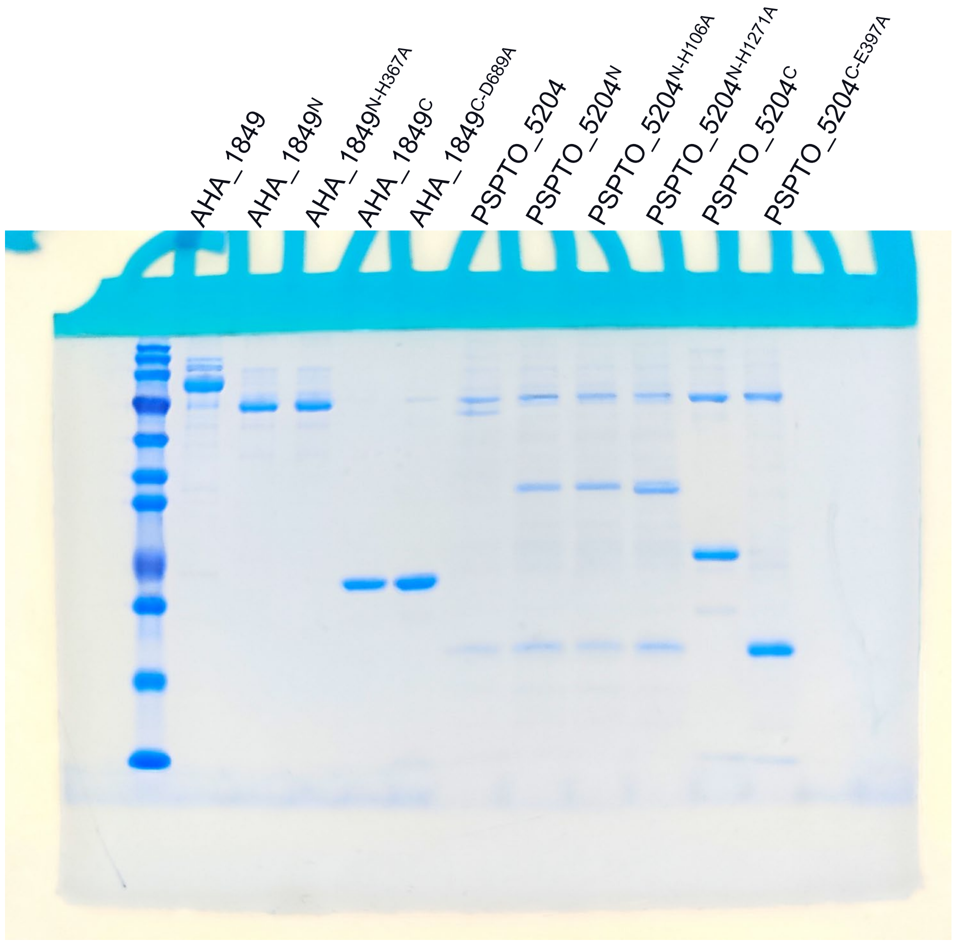

Figure 7-figure supplement 3D, Purification of AHA_1849, PSPTO_5204, and their mutants. Proteins were used for PG-digestion analysis in Figure 4B and 4C.

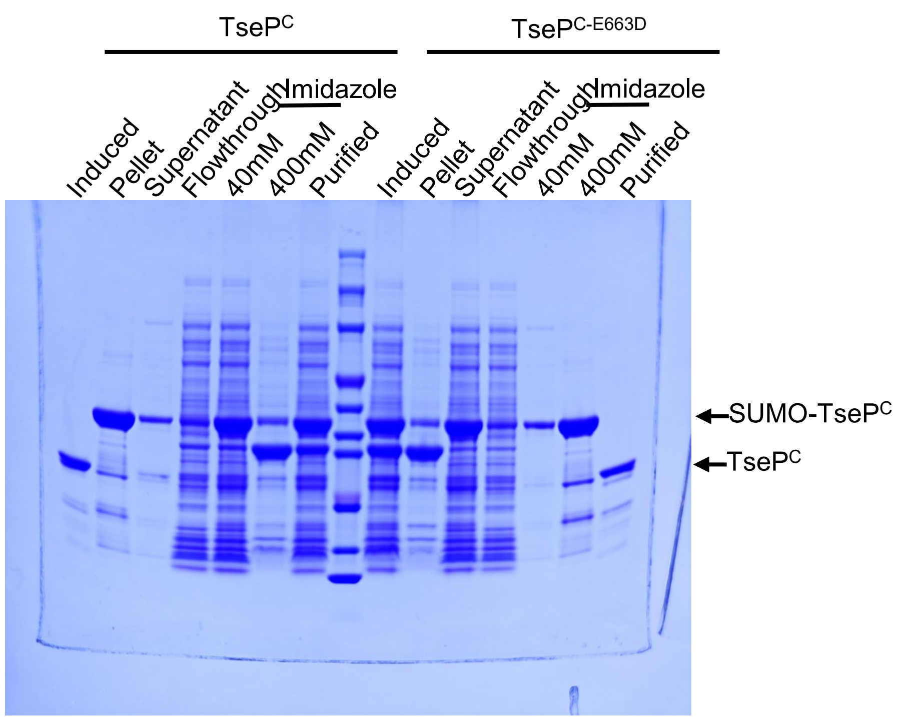

Figure 7-figure supplement 3E, Purification of TseP^{C} and TseP$^{C\text{-}E663D}$ with an His-SUMO tag. The His-SUMO tag was removed by SUMO protease.

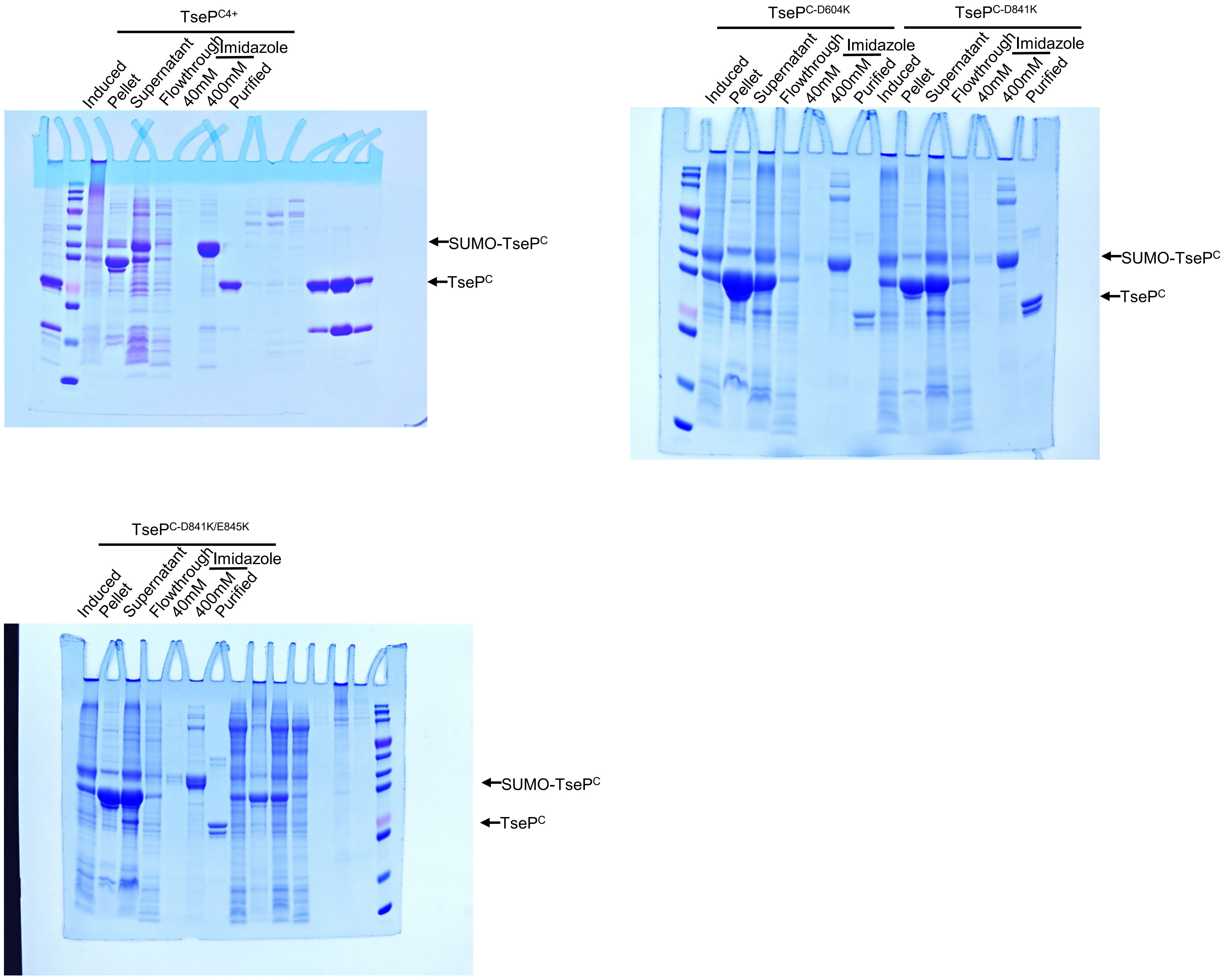

Figure 7-figure supplement 3F, Purification of $TseP^{C}$ variants with an His-SUMO tag. The His-SUMO tag was removed by SUMO protease.
