# Supplementary material for: Amidase and lysozyme dual functions in TseP reveal a new family of chimeric effectors in the type VI secretion system
Source: eLife. 2025 Mar 10;13:RP101125. doi: 10.7554/eLife.101125 (PMC11893102; doi:10.7554/eLife.101125)
Supplement: Supplementary file 1. — (A) Strains and plasmids used in this study. (B) The sequence identity of the N-terminal of TseP and homologs. Sequence identity was calculated using ClustalW web server. Colors are assigned from blue to red, indicating low to high sequence similarity, respectively. (C) The sequence identity of the C-terminal of TseP and homologs. Sequence identity was calculated using ClustalW web server. Colors are assigned from blue to red, indicating low to high sequence similarity, respectively. [file elife-101125-supp1.docx]

**Supplementary file 1a.** Strains and plasmids used in this study.

| **Strain** | **Genotype** | **Description** | | **Source** |
| --- | --- | --- | --- | --- |
| *E. coli* |  |  |  | |
| BL21(DE3) | *F– ompT gal dcm lon hsdSB(rB–mB–) λ(DE3 [lacI lacUV5T7p07 ind1 sam7 nin5]) [malB+]K-12(λS)* | Strain used for protein expression | Lab stock | |
| DH5α | *F- Φ80lacZΔM15 Δ(lacZYA-argF) U169 recA1 endA1 hsdR17 phoA supE44 thi-1 gyrA96 relA1 λ-* | Strain used for cloning | Invitrogen | |
| T-fast | *F' proA+ B+ lacIq ∆lacZM15 / fhuA2 ∆(lac-proAB) glnV galK16 galE15 R(zgb-210::Tn10)TetR endA1 thi-1 ∆(hsdS-mcrB)5* | Strain used for cloning | TIANGEN | |
| MG1655 | *F-*, *lambda-, rph-1* | K-12 wild-type strain used as prey for T6SS killing | Lab stock | |
| *Aeromonas dhakensis* SSU | WT | Parental strain | ^[1]^ | |
|  | Δ*vasK* | T6SS null, in-frame deletion of *vasK* | ^[1]^ | |
|  | Δ*tseP* | In-frame deletion of *tseP* | ^[1]^ | |
|  | Δ*3eff*, *vipA*-*sfgfp* | In-frame deletion of *tseP*, *tseI*, and *tseC*, and fusion expression of sfGFP protein and VipA protein. | This study | |

|  | **Plasmid** | **Description** | **Source** |
| --- | --- | --- | --- |
| pBAD24 | pBAD24Kan | Arabinose inducible expression plasmid, kanamycin resistance | Lab stock |
|  | pBAD24kan-VgrG2-TseP-V5 | Arabinose inducible expression of VgrG2 and TseP | This study |
|  | pBAD24kan-VgrG2-TseP^N^-V5 | Arabinose inducible expression of VgrG2 and TseP^N^ | This study |
|  | pBAD24kan-VgrG2-TseP^C^-V5 | Arabinose inducible expression of VgrG2 and TseP^C^ | This study |
|  | pBAD24kan-VgrG2-TseP^H19A^-V5 | Arabinose inducible expression of VgrG2 and TseP ^H19A^ | This study |
|  | pBAD24kan-VgrG2-TseP^H23A^-V5 | Arabinose inducible expression of VgrG2 and TseP^H23A^ | This study |
|  | pBAD24kan-VgrG2-TseP^H109A^-V5 | Arabinose inducible expression of VgrG2 and TseP^H109A^ | This study |
|  | pBAD24kan-VgrG2-TseP^H339A^-V5 | Arabinose inducible expression of VgrG2 and TseP^H339A^ | This study |
|  | pBAD24kan-VgrG2-TseP^H359A^-V5 | Arabinose inducible expression of VgrG2 and TseP^H359A^ | This study |
|  | pBAD24kan-VgrG2-TseP^E539A^-V5 | Arabinose inducible expression of VgrG2 and TseP^E539A^ | This study |
|  | pBAD24kan-VgrG2-TseP^EFhandmut^-V5 | Arabinose inducible expression of VgrG2 and TseP^EFhandmut^ | This study |
| pBAD18 | pBAD18Cm | Arabinose inducible expression plasmid, choloramphenicol resistance | Lab stock |
| pET28a |  | IPTG inducible expression plasmid, gentamycin resistant | Lab stock |
|  | pET28a-His-SUMO-TseP | IPTG inducible expression of SUMO-TseP fusion with an N-terminal 6×His tag | Lab stock |
|  | pET28a-His-SUMO-TseP^E663A^ | IPTG inducible expression of SUMO-TseP^E663A^ fusion with an N-terminal 6×His tag | ^[2]^ |
|  | pET28a-His-SUMO-TseP^N^ | IPTG inducible expression of SUMO-TseP fusion with an N-terminal 6×His tag | This study |
|  | pET28a-His-SUMO-TseP^N-H19A^ | IPTG inducible expression of SUMO-TseP^N-H19A^ fusion with an N-terminal 6×His tag | This study |
|  | pET28a-His-SUMO-TseP^N-H23A^ | IPTG inducible expression of SUMO-TseP^N-H23A^ fusion with an N-terminal 6×His tag | This study |
|  | pET28a-His-SUMO-TseP^N-H109A^ | IPTG inducible expression of SUMO-TseP^N-H109A^ fusion with an N-terminal 6×His tag | This study |
|  | pET28a-His-SUMO-TseP^N-H339A^ | IPTG inducible expression of SUMO-TseP^N-H339A^ fusion with an N-terminal 6×His tag | This study |
|  | pET28a-His-SUMO-TseP^N-H359A^ | IPTG inducible expression of SUMO-TseP^N-H359A^ fusion with an N-terminal 6×His tag | This study |
|  | pET28a-His-SUMO-TseP^N-E539A^ | IPTG inducible expression of SUMO-TseP^N-E539A^ fusion with an N-terminal 6×His tag | This study |
|  | pET28a-His-SUMO-TseP^C^ | IPTG inducible expression of SUMO-TseP^C^ fusion with an N-terminal 6×His tag | This study |
|  | pET28a-His-SUMO-TseP^C-E663D^ | IPTG inducible expression of SUMO-TseP^C-E663D^ fusion with an N-terminal 6×His tag | This study |
|  | pET28a-His-SUMO-TseP^C4+^ | IPTG inducible expression of SUMO-TseP^C4+^ fusion with an N-terminal 6×His tag | This study |
|  | pET28a-His-SUMO-TseP^C-D604K^ | IPTG inducible expression of SUMO-TseP^C-D604K^ fusion with an N-terminal 6×His tag | This study |
|  | pET28a-His-SUMO-TseP^C-D841K^ | IPTG inducible expression of SUMO-TseP^C-D841K^ fusion with an N-terminal 6×His tag | This study |
|  | pET28a-His-SUMO-TseP^C-D841K/E845K^ | IPTG inducible expression of SUMO-TseP^C-D841K/E845K^ fusion with an N-terminal 6×His tag | This study |
|  | pET28a-His-SUMO-TsiP | IPTG inducible expression of SUMO-TsiP fusion with an N-terminal 6×His tag | This study |
|  | pET28a-His-SUMO-VgrG2 | IPTG inducible expression of SUMO-VgrG2 fusion with an N-terminal 6×His tag | This study |
|  | pET28a-His-SUMO-AHA_1849 | IPTG inducible expression of SUMO-AHA_1849 fusion with an N-terminal 6×His tag | This study |
|  | pET28a-His-SUMO-AHA_1849^N^ | IPTG inducible expression of SUMO-AHA_1849^N^ fusion with an N-terminal 6×His tag | This study |
|  | pET28a-His-SUMO-AHA_1849^N-H369A^ | IPTG inducible expression of SUMO- AHA_1849^N-H369A^ fusion with an N-terminal 6×His tag | This study |
|  | pET28a-His-SUMO-AHA_1849^C^ | IPTG inducible expression of SUMO-AHA_1849^C^ fusion with an N-terminal 6×His tag | This study |
|  | pET28a-His-SUMO-AHA_1849^C-D689A^ | IPTG inducible expression of SUMO-AHA_1849^C-D689A^ fusion with an N-terminal 6×His tag | This study |
|  | pET28a-His-SUMO-PSPTO_5204 | IPTG inducible expression of SUMO-PSPTO_5204 fusion with an N-terminal 6×His tag | This study |
|  | pET28a-His-SUMO-PSPTO_5204^N^ | IPTG inducible expression of SUMO-PSPTO_5204^N^ fusion with an N-terminal 6×His tag | This study |
|  | pET28a-His-SUMO-PSPTO_5204^N-H106A^ | IPTG inducible expression of SUMO-PSPTO_5204^N-H106A^ fusion with an N-terminal 6×His tag | This study |
|  | pET28a-His-SUMO-PSPTO_5204^N-H271A^ | IPTG inducible expression of SUMO-PSPTO_5204^N-H271A^ fusion with an N-terminal 6×His tag | This study |
|  | pET28a-His-SUMO-PSPTO_5204^C^ | IPTG inducible expression of SUMO-PSPTO_5204^C^ fusion with an N-terminal 6×His tag | This study |
|  | pET28a-His-SUMO-PSPTO_5204^C-E397A^ | IPTG inducible expression of SUMO-PSPTO_5204^C-E397A^ fusion with an N-terminal 6×His tag | This study |

**Supplementary file 1b.** The sequence identity of the N-terminal of TseP and homologs. Sequence identity was calculated using ClustalW web server. Colors are assigned from blue to red indicating low to high sequence similarity, respectively.

|  | 1 | 2 | 3 | 4 | 5 | 6 | 7 | 8 | 9 | 10 | 11 | 12 | 13 | 14 | 15 |
| --- | --- | --- | --- | --- | --- | --- | --- | --- | --- | --- | --- | --- | --- | --- | --- |
| **TseP** |  | 41.8 | 88.2 | 88.7 | 47.1 | 12.4 | 14.3 | 12.1 | 13 | 12.1 | 13.3 | 12.6 | 12.3 | 12.5 | 16.7 |
| NCTC12917_01703 | 41.8 |  | 42.9 | 41.1 | 60.6 | 14.9 | 13.7 | 13 | 14.7 | 13.9 | 13.3 | 10.9 | 10 | 14.4 | 17.4 |
| WP3S18E02_26330 | 88.2 | 42.9 |  | 88.5 | 46.8 | 13 | 12.8 | 10.9 | 15.1 | 12 | 12.8 | 12.4 | 12 | 13.7 | 17 |
| AHA_1849 | 88.7 | 41.1 | 88.5 |  | 47.4 | 12.5 | 11.6 | 12.6 | 13.3 | 11.7 | 13 | 13.1 | 15.7 | 13.1 | 17.1 |
| PLESHI_03291 | 47.1 | 60.6 | 46.8 | 47.4 |  | 14.5 | 13.9 | 13.4 | 16.3 | 12.6 | 13.5 | 11.1 | 14.6 | 13.8 | 17.5 |
| CY652_03435 | 12.4 | 14.9 | 13 | 12.5 | 14.5 |  | 15.6 | 12.8 | 13.5 | 42.1 | 13.2 | 17.4 | 13.5 | 12.2 | 14.9 |
| BTO02_30765 | 14.3 | 13.7 | 12.8 | 11.6 | 13.9 | 15.6 |  | 14 | 13.7 | 13.3 | 18.4 | 13.3 | 13.1 | 34.9 | 12.8 |
| PSPTO_5204 | 12.1 | 13 | 10.9 | 12.6 | 13.4 | 12.8 | 14 |  | 12.3 | 10.7 | 13.2 | 13.2 | 15.3 | 13.4 | 14.6 |
| Dda3937_03232 | 13 | 14.7 | 15.1 | 13.3 | 16.3 | 13.5 | 13.7 | 12.3 |  | 13.3 | 17.2 | 14.2 | 14.4 | 17.4 | 18.1 |
| EBL_c11670 | 12.1 | 13.9 | 12 | 11.7 | 12.6 | 42.1 | 13.3 | 10.7 | 13.3 |  | 13.7 | 12.3 | 10.7 | 11.6 | 12.8 |
| SAMN04488136_12163 | 13.3 | 13.3 | 12.8 | 13 | 13.5 | 13.2 | 18.4 | 13.2 | 17.2 | 13.7 |  | 11.8 | 13.5 | 17.3 | 14.9 |
| ABW06_23985 | 12.6 | 10.9 | 12.4 | 13.1 | 11.1 | 17.4 | 13.3 | 13.2 | 14.2 | 12.3 | 11.8 |  | 11.6 | 12.4 | 12.2 |
| SAMN05216206_1427 | 12.3 | 10 | 12 | 15.7 | 14.6 | 13.5 | 13.1 | 15.3 | 14.4 | 10.7 | 13.5 | 11.6 |  | 15.9 | 16.3 |
| C4K68_12090Tola_1753 | 12.5 | 14.4 | 13.7 | 13.1 | 13.8 | 12.2 | 34.9 | 13.4 | 17.4 | 11.6 | 17.3 | 12.4 | 15.9 |  | 13.8 |
| Tola_1753 | 16.7 | 17.4 | 17 | 17.1 | 17.5 | 14.9 | 12.8 | 14.6 | 18.1 | 12.8 | 14.9 | 12.2 | 16.3 | 13.8 |  |

**Supplementary file 1c.** The sequence identity of the C-terminal of TseP and homologs. Sequence identity was calculated using ClustalW web server. Colors are assigned from blue to red indicating low to high sequence similarity, respectively.

|  | 1 | 2 | 3 | 4 | 5 | 6 | 7 | 8 | 9 | 10 | 11 | 12 | 13 | 14 | 15 |
| --- | --- | --- | --- | --- | --- | --- | --- | --- | --- | --- | --- | --- | --- | --- | --- |
| **TseP** |  | 10 | 11.8 | 12.4 | 13.6 | 48.1 | 48.9 | 49.8 | 20.7 | 24.2 | 19.8 | 17.4 | 10.1 | 19.3 | 12.1 |
| NCTC12917_01703 | 10 |  | 69.2 | 18.7 | 11.4 | 9.5 | 11.4 | 11.4 | 11.8 | 9.5 | 11.4 | 11.4 | 14.7 | 12.3 | 17.1 |
| WP3S18E02_26330 | 11.8 | 69.2 |  | 17.1 | 11.3 | 12.2 | 10.9 | 11.8 | 11.3 | 10.9 | 11.8 | 10 | 14.9 | 11.8 | 15.4 |
| AHA_1849 | 12.4 | 18.7 | 17.1 |  | 12.4 | 10.9 | 10.4 | 14 | 11.4 | 11.4 | 10.9 | 12.4 | 15 | 12.4 | 13.5 |
| PLESHI_03291 | 13.6 | 11.4 | 11.3 | 12.4 |  | 14.2 | 14 | 14.1 | 13.9 | 13.1 | 13.2 | 13 | 13.6 | 14.6 | 11.6 |
| CY652_03435 | 48.1 | 9.5 | 12.2 | 10.9 | 14.2 |  | 46.4 | 49.8 | 21 | 23.6 | 19.8 | 20.6 | 11.4 | 21.5 | 11.6 |
| BTO02_30765 | 48.9 | 11.4 | 10.9 | 10.4 | 14 | 46.4 |  | 50.6 | 21.3 | 22.6 | 20.7 | 20.4 | 10.1 | 22.7 | 9.8 |
| PSPTO_5204 | 49.8 | 11.4 | 11.8 | 14 | 14.1 | 49.8 | 50.6 |  | 22.4 | 24.6 | 20.3 | 17 | 10.5 | 21 | 10.3 |
| Dda3937_03232 | 20.7 | 11.8 | 11.3 | 11.4 | 13.9 | 21 | 21.3 | 22.4 |  | 58.5 | 26.4 | 21.3 | 10.1 | 33.9 | 9.4 |
| EBL_c11670 | 24.2 | 9.5 | 10.9 | 11.4 | 13.1 | 23.6 | 22.6 | 24.6 | 58.5 |  | 25.1 | 23.3 | 10.1 | 35.2 | 8.5 |
| SAMN04488136_12163 | 19.8 | 11.4 | 11.8 | 10.9 | 13.2 | 19.8 | 20.7 | 20.3 | 26.4 | 25.1 |  | 21.1 | 9.7 | 25.1 | 10.3 |
| ABW06_23985 | 17.4 | 11.4 | 10 | 12.4 | 13 | 20.6 | 20.4 | 17 | 21.3 | 23.3 | 21.1 |  | 12.7 | 18.9 | 10.7 |
| SAMN05216206_1427 | 10.1 | 14.7 | 14.9 | 15 | 13.6 | 11.4 | 10.1 | 10.5 | 10.1 | 10.1 | 9.7 | 12.7 |  | 11 | 50.4 |
| C4K68_12090Tola_1753 | 19.3 | 12.3 | 11.8 | 12.4 | 14.6 | 21.5 | 22.7 | 21 | 33.9 | 35.2 | 25.1 | 18.9 | 11 |  | 11.6 |
| Tola_1753 | 12.1 | 17.1 | 15.4 | 13.5 | 11.6 | 11.6 | 9.8 | 10.3 | 9.4 | 8.5 | 10.3 | 10.7 | 50.4 | 11.6 |  |

**References**

[1] X. Liang, R. Moore, M. Wilton, M. J. Q. Wong, L. Lam, and T. G. Dong. Identification of divergent type VI secretion effectors using a conserved chaperone domain. Proc. Natl. Acad. Sci. U. S. A. 2015, 112(29):9106–9111.

[2] X. Liang, T.-T. Pei, Z.-H. Wang, W. Xiong, L.-L. Wu, P. Xu, S. Lin, and T. G. Dong. Characterization of lysozyme-like effector TseP reveals the dependence of type VI secretion system (T6SS) secretion on effectors in *Aeromonas dhakensis* strain SSU. Appl. Environ. Microbiol. 87(12):e00435-21.

[3] X. Liang, T.-T. Pei, H. Li, H.-Y. Zheng, H. Luo, Y. Cui, M.-X. Tang, Y.-J. Zhao, P. Xu, and T. Dong. VgrG-dependent effectors and chaperones modulate the assembly of the type VI secretion system. PLOS Pathog. 2021, 17(12):e1010116.
